# Airborne transmission may have played a role in the spread of 2015 highly pathogenic avian influenza outbreaks in the United States

**DOI:** 10.1038/s41598-019-47788-z

**Published:** 2019-08-13

**Authors:** Yang Zhao, Brad Richardson, Eugene Takle, Lilong Chai, David Schmitt, Hongwei Xin

**Affiliations:** 1 Present Address: Department of Agricultural and Biological Engineering, Mississippi State University, Mississippi State, MS 39762 USA; 2 Department of Agricultural and Biosystems Engineering, Iowa State University, Ames, IA 50011 USA; 3 Department of Agronomy, Iowa State University, Ames, IA 50011 USA; 4 Present Address: Department of Poultry Science, University of Georgia, Athens, GA 30602 USA; 5 Iowa Department of Agriculture and Land Stewardship, Des Moines, IA 50319 USA; 6 Present Address: The University of Tennessee Institute of Agriculture, The University of Tennessee, Knoxville, TN 37996 USA

**Keywords:** Air microbiology, Pathogens

## Abstract

The unprecedented 2015 outbreaks of highly pathogenic avian influenza (HPAI) H5N2 in the U.S. devastated its poultry industry and resulted in over $3 billion economic impacts. Today HPAI continues eroding poultry operations and disrupting animal protein supply chains around the world. Anecdotal evidence in 2015 suggested that in some cases the AI virus was aerially introduced into poultry houses, as abnormal bird mortality started near air inlets of the infected houses. This study modeled air movement trajectories and virus concentrations that were used to assess the probability or risk of airborne transmission for the 77 HPAI cases in Iowa. The results show that majority of the positive cases in Iowa might have received airborne virus, carried by fine particulate matter, from infected farms within the state (i.e., intrastate) and infected farms from the neighboring states (i.e., interstate). The modeled airborne virus concentrations at the Iowa recipient sites never exceeded the minimal infective doses for poultry; however, the continuous exposure might have increased airborne infection risks. In the worst-case scenario (i.e., maximum virus shedding rate, highest emission rate, and longest half-life), 33 Iowa cases had > 10% (three cases > 50%) infection probability, indicating a medium to high risk of airborne transmission for these cases. Probability of airborne HPAI infection could be affected by farm type, flock size, and distance to previously infected farms; and more importantly, it can be markedly reduced by swift depopulation and inlet air filtration. The research results provide insights into the risk of airborne transmission of HPAI virus via fine dust particles and the importance of preventative and containment strategies such as air filtration and quick depopulation of infected flocks.

## Introduction

The devastating outbreaks of highly pathogenic avian influenza (HPAI) in the U.S. in 2015 caused significant economic losses to the poultry industry and disruption of egg supply (shortage and price hike). In less than 7 months, 232 farms in 15 states and over 50 million birds were affected^1^, with a total economic loss valued at $3.3 billion^2^. The outbreak was eventually under control after massive culling; but how the disease was spread among the farms remains unknown. As traditional biosecurity protocols for preventing direct contact and vector transmissions proved unsuccessful, airborne transmission – a mechanism by which virus is carried in fine particulate matter (PM) and aerially transported from infected to recipient farms – received increasing attention and concern. Anecdotal evidence in 2015 claimed that bird mortality started to increase near the air inlets of the inflected poultry houses. Recent studies showed that AI virus was airborne transmissible in a ferret model^3,4^. Some vigilant producers have installed or planned to install incoming air-treatment systems to reduce the risk of airborne transmission of AI virus, even though such transmission pathway remains scientifically explored. This is the essence and motivation of our study described in this paper – investigating the risk of AI airborne transmission by making use of the 2015 HPAI data and offering science-based guidance for implementing effective prevention or containment strategies^5^.

Efforts have been made to recover airborne AI virus downwind of infected farms by using aerosol samplers, as done during the 2015 outbreak^6^. While air sampling is a direct method to investigate local and regional transmissions^7^, it is not suitable for delineating long-distance transmission because of its inherent limitations such as insensitivity of the samplers^8^, sampling stress imposed to target pathogens^9^, and difficulty in selecting sampling locations due to varying wind direction. As such, long-distance transmission of pathogens in large scale has been often examined with various meteorological models. Among the various models, the Hybrid Single Particle Lagrangian Integrated Trajectory (HYSPLIT) model is frequently used for animal pathogens like foot-and-mouth disease virus^10,11^, blue tongue virus^12^, etc. The major advantages of the HYSPLIT model lie in its flexibility to almost all inhomogeneous and time-varying meteorological conditions as well as non-flat terrain^13^, and its ability to project backward trajectories and visualize origins of viral particles^14^. As an incorporated module, it accounts for biological decay of pathogens under different environmental conditions that improves the modeling accuracy^11^. Air trajectory and concentration modelings are the core modules of the HYSPLIT model.

Modeling studies of airborne transmission of AI virus among farms are scant. Of the limited published studies^15,16^, all dealt with cases within small areas (5–25 km radius). However, the airborne viral particles could be transported over hundreds of kilometers, thus resulting in much broader impacts^17,18^. To address the long-distance airborne transmission of HPAI, we collected information of all 232 infected cases spanning 15 states in the U.S. during the 2015 HPAI outbreak, and examined airborne transmission of AI virus from these cases to the 77 infected cases in Iowa (included in the 232 total cases). We modeled the air movement trajectories from the previously infected farms to the recipient farms, and estimated virus concentrations that were used to assess the probability of airborne transmission for the Iowa cases. Factors affecting the probability of airborne transmission were investigated. We also examined the effectiveness of airborne transmission risk-reduction strategies, including swift depopulation of the infected flocks (within 24 h ) and filtration of incoming ventilation air at the recipient farms. While this study focuses on airborne transmission only, it is not our intention to rule out any other possible transmission modes, e.g. direct, vector, or combined transmissions.

## Materials and Methods

### Infected poultry farms

Information of 232 infected poultry farms was obtained from the Emergency Management Response System (EMRS) of USDA Animal and Plant Health Inspection Service (APHIS). Each record or infected farm was identified by farm name, physical address, county and state, infection confirmation date, depopulation date, and number of birds infected. In this study, we focused on the intra-state and inter-state airborne transmission for all 77 cases in Iowa (Table S3). Only infected farms corresponding to the Iowa outbreak period (3/22/2015-6/15/2015) were included in the modeling. The reason for focusing on Iowa cases is that they accounted for the majority of the bird loss (65% of the national total) and included a mixture of turkeys and laying hens (majority of the birds lost).

### HYSPLIT modeling

Backward trajectory modeling was performed for Iowa cases to examine if they received air passing other infected farms before they became infected. Considering the incubation period could be as long as 21 days (communication with Dr. James Roth at Iowa State University), air trajectories of 21 days prior to the infection confirmation date were investigated for each case. As temporal wind speed and direction varied, modeling was done at 8-hr time intervals on each day, yielding three trajectories covering the air arrival time at 8 am, 4 pm and 12 am Local Standard Time (LST). Incoming air to the recipient farms was examined at the height of 1.5 m above ground for turkey farms and 3.0 m for hen farms, considering the height of the typical air inlets. These heights were considered because air entered most turkey growing houses through ventilation curtains surrounding side walls while air entered typical hen houses through eave inlets. Backyard farms were treated as turkey farms, and pullets (young hens before sexual maturity) farms were treated as hen farms. The trajectory was exported as.kmz file, and loaded in Google Earth for further analysis. With backward trajectory modeling, we projected air movements showing clear patterns that viral particles could have been transported from originating infected farms to recipient farms. This finding necessitated concentration modeling to look at whether the amounts of virus released from the infected farms were high enough to cause infections at the recipient farms.

The concentrations of airborne virus carried by PM_10_ (PM with aerodynamic diameter smaller than 10 µm) and PM_2.5_ (PM with aerodynamic diameter smaller than 2.5 µm) were estimated using both default and ceiling input values in the HYSPLIT model (Table S1). These two PM sizes have received increasing concerns of public health in the past two decades as they are small to travel long distances and deposit in lower airways of recipients^19,20^, thus more readily inducing respiratory problems. The default input values were the most representative data from scientific references; however, extreme/worst-case scenarios might have occurred when the values of some input parameters were at the ceiling values. Virus concentration modeling was performed on a daily basis. Two concentration contour maps, one for virus carried by PM_10_ and the other by PM_2.5_, were developed for each day during the outbreak period. We focused on the concentrations at 0–6 m above the ground to reflect the height of most poultry facilities. Parameters required for the modeling were either obtained from our own measurements or from scientific references (Table S1). The HYSPLIT model outputs a series of 12-km-apart virus concentration contours. The virus concentration at a farm was the mean concentration value of two consecutive contours that envelop the farm of concern. The modeled virus concentrations were compared with the minimal infective doses (MIDs) for poultry and were used to determine the probability of infection for each Iowa case.

### Minimal infective dose

The MIDs were calculated based on the infective dose, lung capacity, respiratory rate, and exposure time. The infective doses were 10^3^ 50% egg infective dose (EID_50_) for turkeys^21^ and 10^3.5^ EID_50_ for laying hens^22^. Lung capacity and respiratory rate are 7.7 × 10^−5^ m^3^ and 2.4 × 10^3^ times/h for turkeys, and 1.4 × 10^−5^ m^3^ and 1.6 × 10^3^ times/h for laying hens, respectively^23,24^. The resultant MIDs were 10 EID_50_/m^3^ for turkeys and 281 EID_50_/m^3^ for laying hens over a 21-day exposure.

### Calculation of infection probability

A binomial distribution was used to calculate the probability of farm infection, as described in previous studies^11,25^. The probability of farm infection (*P*_*h*_) depends on the probability of individual-animal infection (*P*_*i*_) at a certain dosage (*d*) and flock size (*n*_*f*_). The probability that one EID_50_ of AI virus will infect an animal (*θ*) has not been reported. By conservatively assuming 95% of birds would get infected at the aforementioned infective doses, *θ* was 0.00069 for turkeys and 0.00022 for laying hens.

1 $${P}_{i}=1-{(1-\theta )}^{d}$$

2 $${P}_{h}=1-{(1-{P}_{i})}^{{n}_{f}}$$

To take into account that a flock may be exposed for multiple days (*n*_*d*_), we used the following formula.

3 $${P}_{h}=1-{\prod }_{i=1}^{{n}_{d}}{(1-{\theta }_{i})}^{{d}_{i}\cdot {n}_{f}}$$

## Results

### Backward air trajectories to recipient farms in Iowa

**Figure 1:**
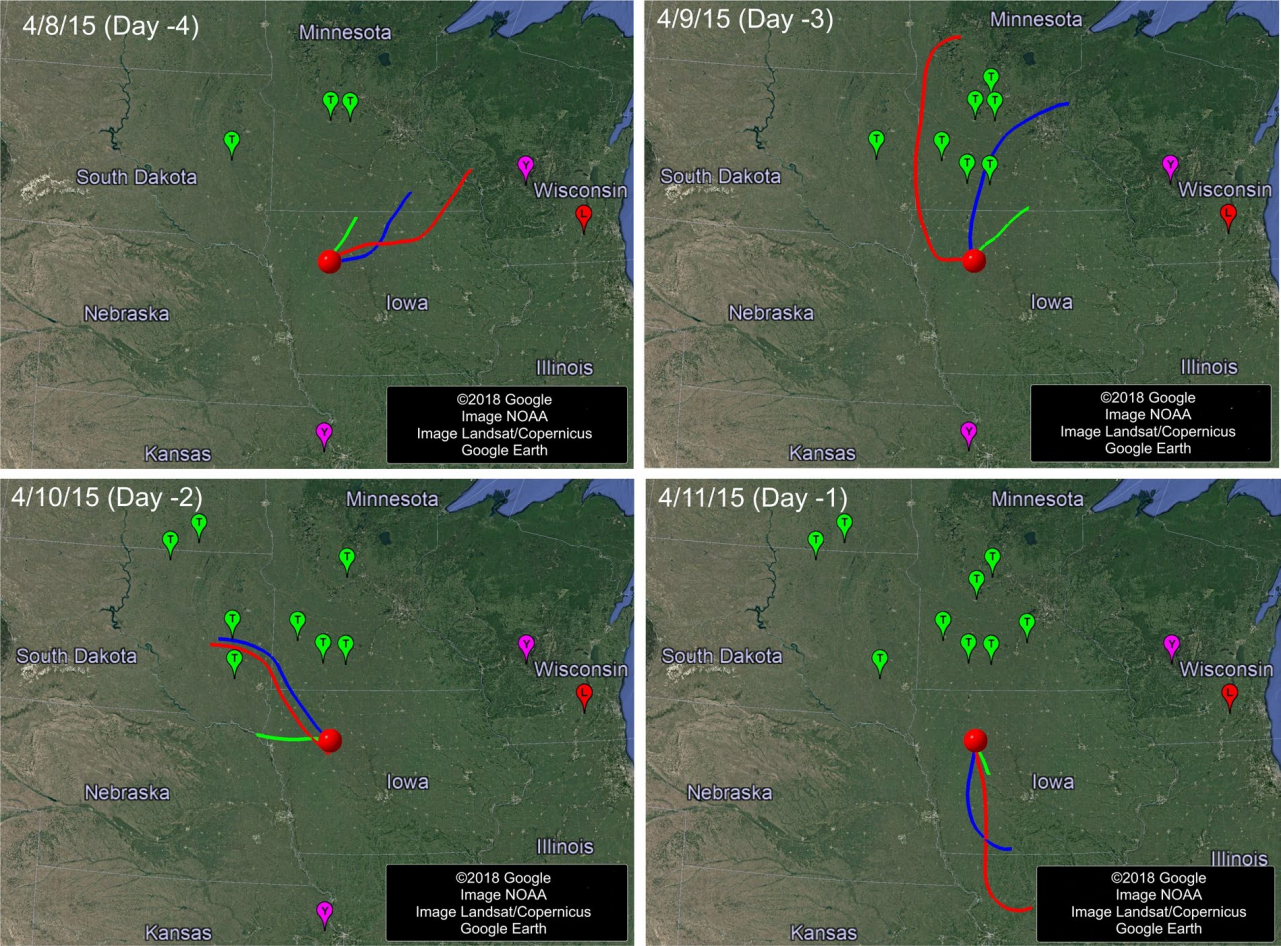
Backward air trajectories of four example days (4/8 – 4/11/2015) prior to the infection confirmation date (4/12/2015) of the first Iowa case. Red, blue and green lines are the 24-h (12am-12am, Local Standard Time or LST), 16-h (12am-4pm, LST) and 8-h (12am-8am, LST) trajectories, respectively. The round red dot represents the first case location; other dots are cases (green for turkey, red for laying hen, purple for backyard) that had been confirmed positive before the first Iowa case and the infected flocks had not yet been depopulated.

**Figure 2:**
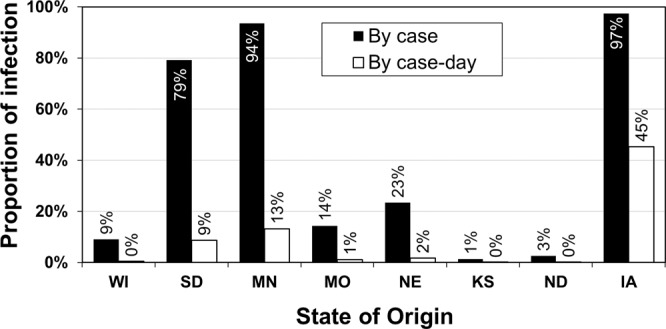
Proportions of HPAI infected cases and case-days in Iowa that were possibly attributable to airborne transmission. The total number of Iowa cases is 77, and the total number of case-days is 1,617 (77 cases, each was examined 21 days prior to the date of infection confirmation).

Figure 1 shows four example days of trajectories from 4/8/2015 to 4/11/2015 for the first Iowa case, which was confirmed on 4/12/2015. The area surrounded by the three 8-hour air trajectories covers the infected farms in Minnesota confirmed on 4/9/2015 and those in South Dakota confirmed on 4/10/2015, indicating that the first Iowa case could have received airborne AI virus from these two nearby states and the infection might have occurred two to three days before the confirmation date. The same analysis was done for the remaining 17 days (3/22/2015–4/7/2015) of this Iowa case, and the process was repeated for the rest of Iowa cases. The states as the potential AI sources and possible infection day for each Iowa case are listed in Table S2. Air trajectory modeling shows that 97% of the Iowa cases or 45% of the Iowa case-days could have received virus-laden air from previously infected farms within Iowa, deemed as potential intra-state airborne transmission (Fig. 2). Airborne virus could also come from seven nearby states at different degrees of likelihood, including Minnesota (94% by case and 13% by case-day, or 94%/13%), South Dakota (79%/9%), Nebraska (23%/2%), Missouri (14%/1%), Wisconsin (9% /< 1%), North Dakota (3% /< 1%), and Kansas (1% /< 1%).

### Virus concentration and infection risk of AI airborne transmission: *based on default values of input parameters*

**Figure 3:**
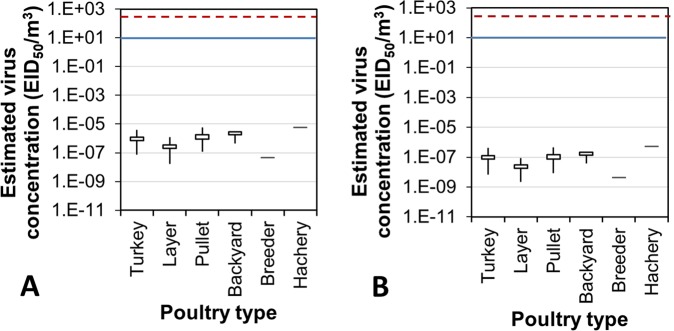
Ninety-five percent confidence interval (95% CI), maximal and minimal concentrations of airborne avian influenza virus carried by PM_10_ (**A**) and PM_2.5_ (**B**) at the infected farms in Iowa. Concentrations were estimated based on the default values. Top and bottom horizontal lines of each rectangular box are the upper and lower limits of 95% CI, respectively. Free ends of the top and bottom vertical lines are the maximal and minimal virus concentrations, respectively. Red dash line and blue solid line are the MIDs for laying hen and turkey, respectively.

Based on the default values of the model input parameters (Table S1), the 21-day mean airborne virus concentrations experienced by the Iowa cases were well below 10^−4^ EID_50_ (50% egg infective dose)/m^3^ (Fig. 3). These levels were all lower than the MID for either turkeys or laying hens on any day during the outbreak period in Iowa. On average, airborne virus concentrations for egg layer and breeder farms seemed to be lower than those for turkey, pullet, backyard, and hatchery farms. The concentrations of virus carried by PM_10_ were approximately 10 times those carried by PM_2.5_. Similarly, the impact area (with virus concentration higher than 10^−7^ EID_50_/m^3^) of PM_10_ was much greater than that of PM_2.5_ (Movies S1 and S2).

The maximal, average, and minimal probability of airborne infection was, respectively, 3.75%, 0.44% and 0.00% using the viral concentration data of PM_10_; and 0.36%, 0.04% and 0.00% using the viral concentration data of PM_2.5_. There is no commonly accepted clear-cut value for categorization of AI infection risk. Some research on other pathogenic diseases, such as foot-and-mouth, deemed >50%, 10–50%, 1–10% and <1% as high, medium, low and extremely low risk, respectively^11^. Applying these categorical criteria, the infection risk of the Iowa cases by airborne transmission was low to extremely low based on the default model input values.

### Potential virus concentration and infection risk of AI airborne transmission: *based on ceiling values of input parameters*

**Figure 4:**
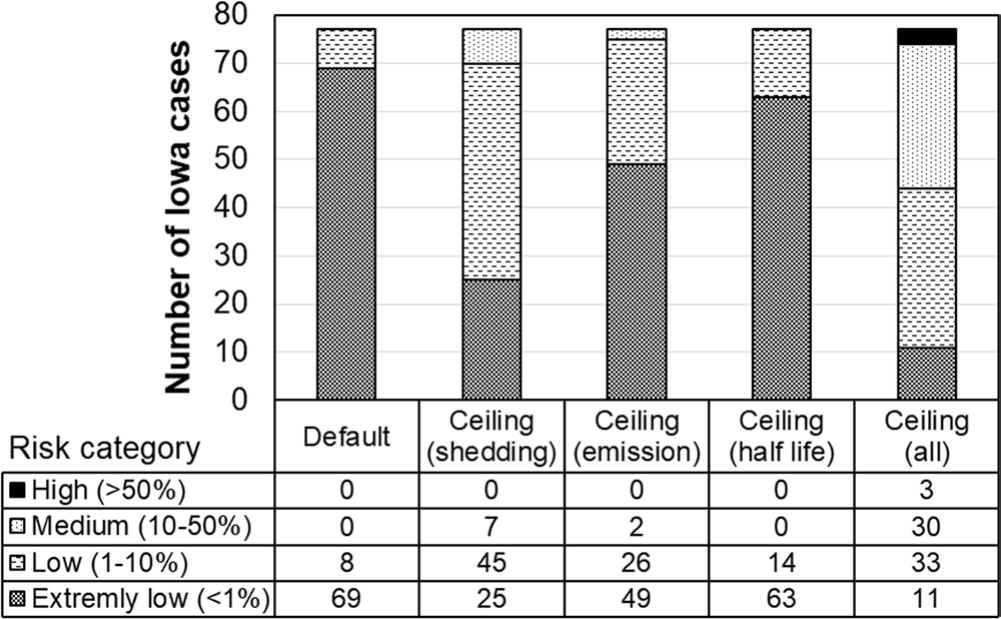
Distribution of Iowa cases at four infection risk categories using default and ceiling values in concentration modeling for virus carried by PM_10_. For ceiling values, distribution was estimated by pushing each parameter to its ceiling value, separately and combined.

Poultry can shed AI virus at a rate of 10^5^ EID_50_/g of feces^26,27^, which is 10-fold the default value. When this shedding rate was used in the model, the infection risk of airborne transmission notably increased, yielding 7 (9%), 45 (58%) and 25 (33%) of the Iowa cases in medium, low, and extremely low risk category, respectively (Fig. 4). The reason that we used fecal, rather than airborne, virus concentration as the model input was because of lack of accurate concentration data for airborne AI virus during the outbreak. Similarly, for virus-laden PM_10_ emission rate and half-life time at 3 and 1.5 times the default values, the risk of airborne transmission was elevated to various levels (Fig. 4). Assuming the worst-case scenario of all three input parameters at peak values, the daily maximal virus concentration carried by PM_10_ reached 1.1 × 10^−3^ EID_50_/m^3^. This virus concentration was still much lower than the poultry MIDs, but it markedly increased the risks of airborne infection. As a result, 3 (4%), 30 (39%), 33 (43%) and 11 (14%) of the Iowa cases were classified in the high, medium, low, and extremely low risk category, respectively (Fig. 4).

**Figure 5:**
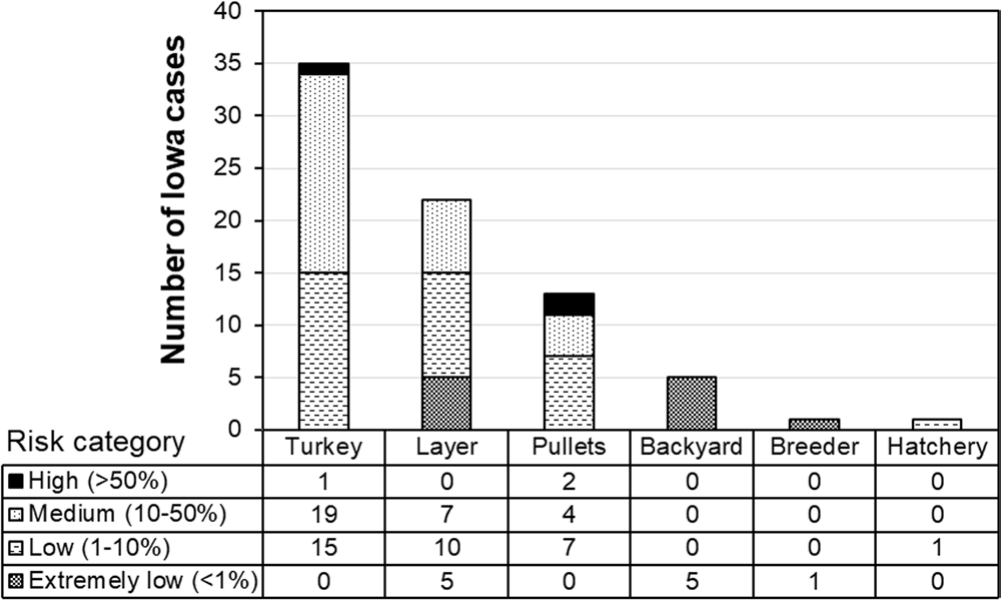
Distribution of Iowa cases at four infection risk categories by farm type. Combined ceiling values of virus shedding rate, emission rate, and half-life are applied.

**Figure 6:**
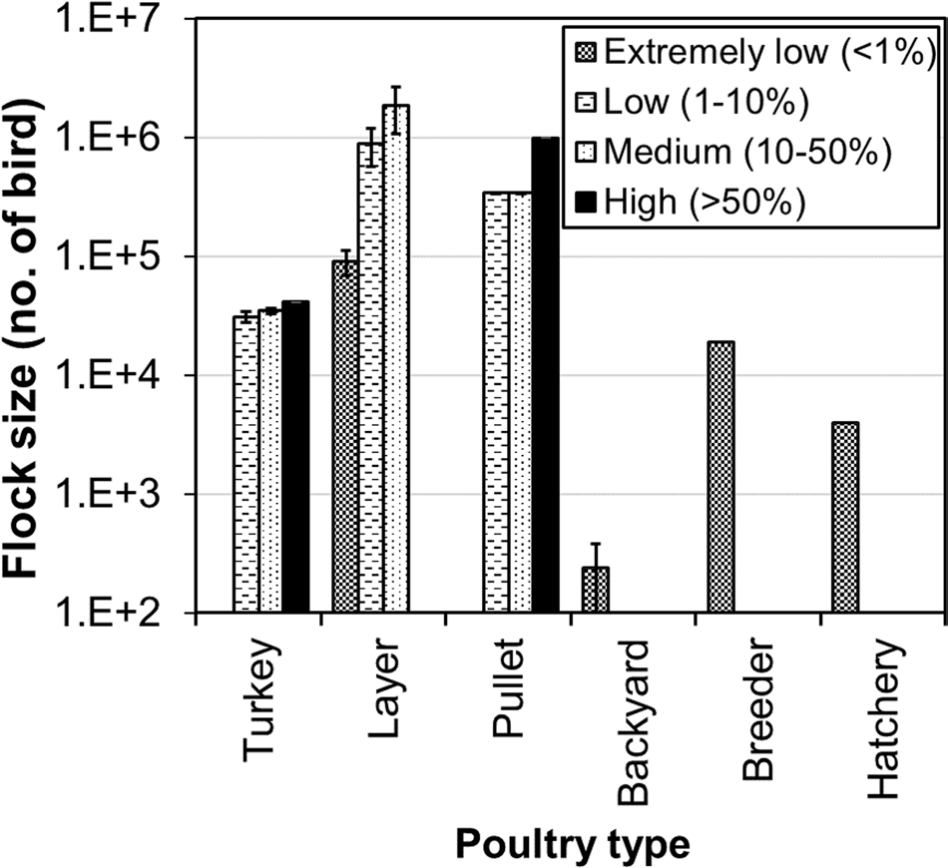
Flock sizes of infected Iowa farms at four infection risk categories. Error bars are 95% confident intervals. Combined ceiling values of virus shedding rate, emission rate, and half-life are applied.

**Figure 7:**
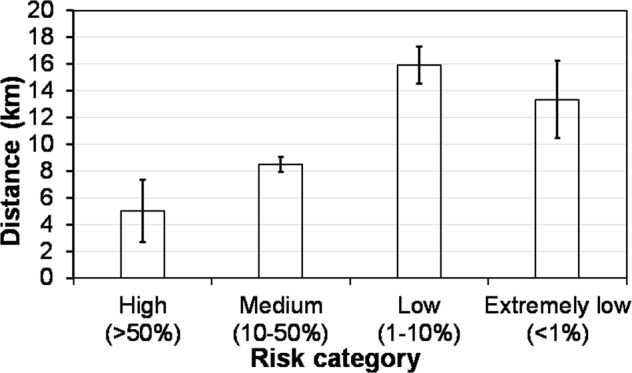
Distance between each Iowa case and its nearest previously infected farm in four airborne infection risk categories. Combined ceiling values of virus shedding rate, emission rate, and half-life were applied. Error bars are 95% CIs.

Under the worst-case scenario, the turkey farms are more vulnerable to airborne transmission (Fig. 5), with 57% farms falling in medium- to high-risk categories, as compared to 32% for the laying-hen farms, 46% for pullet, and 0% for backyard, breeder and hatchery. Figure 6 shows the flock sizes of infected farms at four infection risk categories. For turkey, layer and pullet farms, the probability of airborne infection was higher for larger flocks within the same type of poultry. The nearest average distance to the previously infected cases corresponding to the high, medium, low, and extremely low infection risk category was, respectively, 5.0 km, 8.5 km, 15.9 km, and 13.3 km (Fig. 7). Namely, cases with a closer distance to previous outbreak farm are subject to higher airborne infection risk.

### Reducing airborne transmission risk for the worst-case scenario

**Figure 8:**
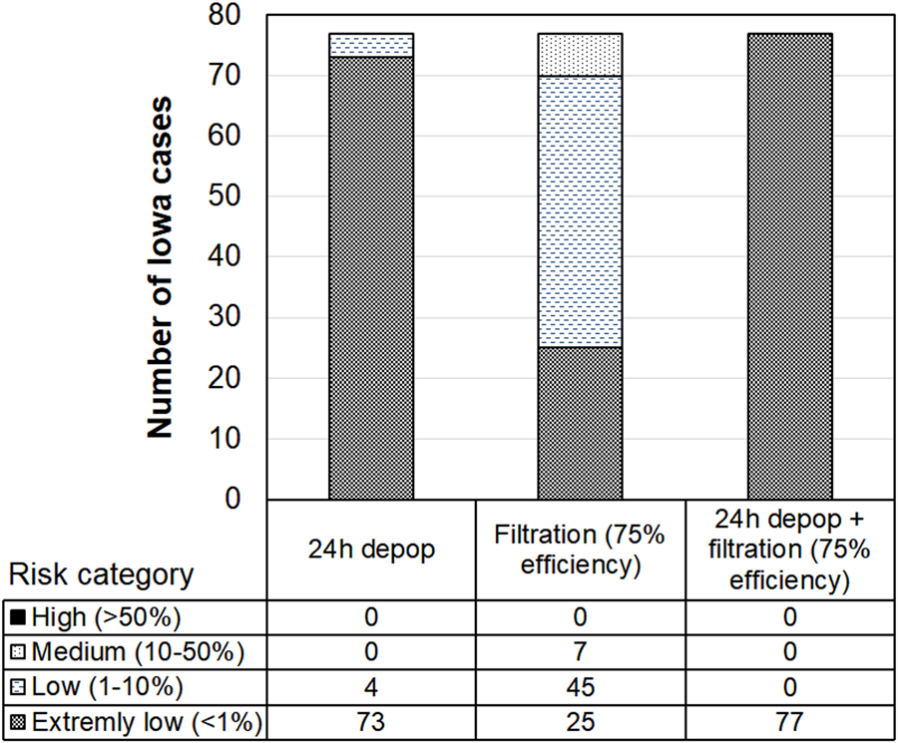
Case distribution at four infection risk categories when applying 24-h depopulation and inlet air filtration strategies to minimize airborne transmission risks. Combined ceiling values of virus shedding rate, emission rate, and half-life for PM_10_ are applied.

The worst-case scenario might have a lower chance of occurrence because some of the parameters could be negatively correlated. For example, emission rate is lower in wintertime due to low ventilation rate^28^ but half-life could be higher because the virus survival favors low temperatures^29,30^. However, it would be prudent to always be prepared for the worst-case scenario to minimize or reduce the risk of such highly contagious disease^31,32^. By means of applying strategies such as fast depopulation of the infected flocks and/or filtration of incoming air, the probability of infection could have been significantly reduced. No farm would fall in medium or high infection risk even for the worst-case scenario (Fig. 8) if depopulation was performed at the infected farms within 24 h. Filtering the incoming air at typical PM reduction efficiency of 75%^33^ would have reduced the risk as well (Fig. 8). A combination (24-h depopulation plus air filtration) strategy would have reduced the airborne risk of all Iowa cases to the extremely low level (Fig. 8).

## Discussion

Our backward air trajectory modeling revealed the possibilities of both intra-state and inter-state airborne transmissions for the Iowa cases. The inter-state transmission was associated only with nearby states (Minnesota, South Dakota, Nebraska, Missouri, Wisconsin, North Dakota, and Kansas). Other states with reported positive cases were excluded from the Iowa cases because the air trajectories either did not pass the sites under consideration or the positive cases ceased to emit virus after depopulation. In this study, air trajectories were projected for 1-day periods because we assumed that the airborne AI virus would significantly lose its viability within this time frame. Though the survival of airborne AI has not been well investigated, studies on human or pig adapted influenza A virus subtypes have revealed pronounced reductions (~10 fold) in their viability in the air within a day^34,35^. Exposure to sunlight and other environmental stress (e.g., variable air humidity and temperature) would further inactivate the influenza virus^36^. Ultimately, the trajectory modeling showed air movements from the originating infected farms to the recipient farms, which warranted concentration modeling for risk analysis.

Previous studies have shown that PM is a carrier of airborne pathogens during transmission^37^. Research has been increasingly focusing on small PM, e.g. PM_10_ and PM_2.5_, because smaller particles, as compared to larger ones, remain airborne for longer time and can deposit deeper in human and animal respiratory systems, thus inducing severer health risk. In this study, the estimated concentrations of virus carried by PM_10_ were much higher than those by PM_2.5_. Although PM_2.5_ may be aerially transported farther due to its lower deposition velocity as compared to PM_10_^38,39^, the amount of virus carried is much less. It has been reported that pathogens carried by a particle is proportional to the particle volume^40^, implying that a spherical particle of 2.5 µm would have only 1.6% holding/carrying capacity of a 10 µm counterpart. As a result, we noticed that the modeled impact area (with virus concentration higher than 10^−7^ EID_50_/m^3^) for PM_10_ was much greater than that for PM_2.5_ (Movies S1 and S2).

Both default and ceiling input values were used for estimating virus concentrations in this study. Although the default values were commonly used in transmission modeling, values leading to higher virus concentrations can exist, e.g., elevated virus emission rate due to higher ventilation rate and longer virus survival under favorable environmental conditions. To avoid underestimating the potential of airborne transmission, the model was run with the ceiling/maximal values for some sensitive parameters including virus shedding rate, emission rate, and half-life. As the amount of virus carried by PM_10_ model was much greater than that for PM_2.5_, we only considered the PM_10_ in modeling with the ceiling values.

Two generally accepted principles govern the infection development in animals. First, infection occurs when an animal receives the amount of pathogen exceeding its MID. Second, a single virus particle is sufficient to infect an animal at a certain probability of infection^25,41^. Our results revealed that the resultant virus concentrations estimated using either default or ceiling input values were far below the poultry MIDs for the 2015 HPAI cases. However, 33 Iowa cases (43% of total) could still have experienced high to medium probability of airborne infection. It suggests that the infection probability model is more sensitive, thus conservative, for assessing airborne infection risk than the MID model.

While virus concentration plays a key role in the infection probability, differences in susceptibility to virus among poultry types matter as well. The MID for turkeys is about one third of that for egg-type birds^21,22^. Furthermore, turkey barns are more exposed to ambient conditions because of the open sidewalls and no air impaction along the pathway; whereas egg-type poultry barns are totally enclosed and employ eave/attic air inlets that can create impaction to incoming particles, thus reducing the amount of particles entering the animal space. These structural differences likely contributed to the facts that more turkey farms were inflected and were prone to higher infection probability than the egg-type poultry facilities.

Other factors affecting probability of infection include flock size and distance to the nearest-infected premises. Larger flocks were found to have greater chance of infection, assuming all birds are equally susceptible to the HPAI virus. Therefore, albeit experiencing similar modeled virus concentrations to the turkey farms, all five infected backyard farms were at low infection risk of airborne transmission because of the small bird flock sizes (Fig. 6). Both plume modeling and onsite air sampling have been performed to determine the virus/particle concentration surrounding source poultry farms^6,16,42^. As distance from a source farm increases, virus/particle concentrations decreased significantly, thus reducing the probability of airborne infection.

As soon as a contagious disease is confirmed at a farm, a regulatory control area will be established surrounding the infected premise. Farms within the control area need to be depopulated or quarantined, and movements of animals and animal products within this area are prohibited or restricted. The minimal size of the control area is 10 km in radius. Our findings reveal that the minimal control area would cover the cases in the high and medium risk categories of airborne infection.

At the beginning of the 2015 HPAI outbreak, the infected farms did not depopulate the flocks immediately upon positive confirmation. Some infected flocks were left on sites to die out naturally, which took weeks (personal communication with an egg producer). This delayed depopulation may have increased the chance of airborne transmission because the HPAI virus kept releasing to ambient environment through ventilation. Based on lessons learned from the 2015 HPAI outbreak, the U.S. Department of Agriculture (USDA) requires that infected birds be depopulated within 24 h of confirmation^43^. Whole-house thermally-assisted ventilation shutdown depopulation (VSD) could be used as a backup plan^44^. Execution of these guidelines might have played a key role in taking fast control of subsequent AI outbreaks in the U.S. Our results also suggested that 24-h depopulation could significantly reduce the probability of infection at the recipient farms. Besides the governmental guidelines for controlling airborne transmission, some egg producers installed or plan to install air filtration systems to reduce the virus particles in the incoming air to prevent AI infection. Our group has been investigating the removal efficiency of an air filtration system with a commercial laying-hen house, and our results to date indicate a reduction rate of up to 75% in incoming particles (depending on the season)^33^. A 75% removal efficiency would help to reduce the infection risk, but 7 cases (9%) are still in medium risk (Fig. 8). To reduce the risk further to low or extremely low, the reduction efficiency of the air inlet filtration system would need to be at least 90%.

While the HYSPLIT model is a useful tool to address potential long-distance airborne transmission of AI virus, it has limitations. Firstly, the horizontal resolution of 12 km makes it less accurate to estimate the virus concentrations within 12 km of an infected farm. Our data show that 44% of the case-days (all in Iowa) had the infected and recipient farms being <12 km apart. For these case-days, even if the infection risk of the recipient farm fell in low-risk category or even if the virus concentration at the recipient farm was lower than the MID, special attention is needed because the virus concentrations could have been underestimated. Secondly, virus is assumed to be evenly distributed in the air, like gas, in the HYSPLIT model. However, the majority of the airborne virus is actually attached to dust particles, resulting in non-uniform distribution of the virus in a microenvironment^45,46^. It is possible that some birds breathe in more viral particles than others^47^. But for infection risk assessment in this study, all birds were assumed to receive or breathe in the same viral dosage. Thirdly, the HYSPLIT model is helpful to understand the possibility and risk of airborne transmission among farms, but an affirmed airborne transmission cannot be concluded without the genetic analysis, e.g. genome sequencing and molecular network analysis^48–50^.

The modeled airborne viral concentrations in Iowa during the 2015 AI outbreak were extremely low (<2.8 × 10^−5^ EID_50_/m^3^). Such viral levels would be extremely challenging, if not impossible, to detect with currently available air samplers. Our attempts to assess airborne transmission by air sampling downwind of three infected farms in Iowa failed isolating any viable virus (Sampler used: Andersen six-stage impactor, all glass ‘AGI-30’ impinger, smart air sampler system ‘SASS’ 3100. Sampling duration: 0.5–2.0 hours. Number of samples: 104). The modeling in the current study suggests that some Iowa cases could have been exposed to high risk of airborne transmission. Thus, air sampling may not be a suitable or reliable method to evaluate long-distance transmission and caution should be taken when interpreting negative air samples because they may not indicate the truly bio-safe aerial environment.

Because the processes of AI airborne transmission – virus shedding, aerosolization, transportation, deposition, and infection – are not fully understood, this study relied on assumptions based on the best available knowledge. We assumed that all virus was only shed through feces, although it is also present in poultry’s respiratory tract and thus may be released through respiration^51,52^. The latter mechanism of AI virus shedding has not been investigated, but some research on other pathogens suggested that the amount may be low^53^. Virus shedding rate of poultry was assumed constant throughout the infected period; however, this shedding rate could vary at different illness stages. After shed in feces, the virus would not be aerosolized immediately due to the relatively high moisture content of fresh feces^54^ that prevents fecal particle from becoming airborne. The ‘latency period’ of aerosolization is an important parameter as it determines the onset of the airborne transmission risk. Studies are lacking that address the latency period directly, although a few reported that this period could be as long as 2 days^53,55^. The biological decay of airborne AI virus remains investigated. Although AI virus could survive for weeks in water or on surfaces, it may lose infectivity much faster in air, probably due to exposure to adverse changes in humidity, temperature, or radiation^56^. Virus survival might also vary with different shedding routes. For example, virus shed through respiratory tract undergoes dehydration stress while virus from feces may undergo both dehydration and rehydration stresses. Information about virus survival responses to the environment stress is lacking. Another vague area is the particle deposition in animal respiratory tract. Depending on the particle size and respiratory mode (nasal vs. oral), the deposition efficiency may vary.

In conclusion, air movement and airborne transmission of HPAI were investigated for 77 Iowa cases of the 2015 outbreak using the HYSPLIT model. During the HPAI outbreak in Iowa, air moved from the infected sites to the recipient sites that were subsequently confirmed positive. The modeled virus concentrations largely depend on the values of the input parameters, such as virus shedding rate, emission rate and half-life. The estimated virus concentrations did not exceed the MIDs for poultry. The infection probability by airborne transmission was generally low, although 33 Iowa cases were at medium to high risk of airborne transmission under the worst-case scenario. The infection risk seems affected by the house type (open sided vs. fully closed), flock size, and distance to the previously infected farm. The risk of AI airborne transmission may be considerably reduced by inlet air filtration and/or fast depopulation of inflected flocks (e.g., within 24 h).

## Supplementary information

Supplementary Information

Movie S1

Movie S2
