# Supplementary material for: Airborne transmission may have played a role in the spread of 2015 highly pathogenic avian influenza outbreaks in the United States
Source: Sci Rep. 2019 Aug 13;9:11755. doi: 10.1038/s41598-019-47788-z (PMC6692305; doi:10.1038/s41598-019-47788-z)
Supplement: Supplementary file 1 — Supplementary Information [file 41598_2019_47788_MOESM1_ESM.docx]

**Supporting information**

**Airborne transmission may have played a role in the spread of 2015 highly pathogenic avian influenza outbreaks in the United States**

**Yang Zhao^1^, Brad Richardson^2^, Eugene Takle^3^, Lilong Chai^2^, David Schmitt^4^, Hongwei Xin^2^**

*^1^ Department of Agricultural and Biological Engineering, Mississippi State University, Mississippi State, MS 39762; ^2^ Department of Agricultural and Biosystems Engineering, Iowa State University, Ames, IA 50011; ^3^ Department of Agronomy, Iowa State University, Ames, IA 50011; and ^4^ Iowa Department of Agriculture and Land Stewardship, Des Moines, IA 50319*

**Table S1. Values of input parameters used for concentration modeling in the HYSPLIT modeling.**

| **Parameter** | **Unit** | **Value** | **Reference** |
| --- | --- | --- | --- |
| Total run time per cycle | h | 24 | - |
|  |  |  |  |
| Trajectory modeling direction | - | Backward | - |
|  |  |  |  |
| Top of the model | m | 1,500 | - |
|  |  |  |  |
| Incubation period | day | 21 | - |
|  |  |  |  |
| Virus emission duration | day | Site dependent | USDA confidential data |
|  |  |  |  |
| Height of concern | m | 0-6 | - |
|  |  |  |  |
| PM_10_ emission rate | mg/bird/d | 10/30 (default/ceiling for hen)  380/1140 (default/ceiling for turkey) | ([1](#_ENREF_1), [2](#_ENREF_2)) |
|  |  |  |  |
| PM_2.5_ emission rate | mg/bird/d | 1 (hen)  38 (turkey) | ([1-3](#_ENREF_1)) |
|  |  |  |  |
| PM density | g/cm^3^ | 1.5 | ([4](#_ENREF_4)) |
|  |  |  |  |
| PM deposition velocity | m/s | 0.001 (PM_2.5_)  0.01 (PM_10_) | ([5](#_ENREF_5)) |
|  |  |  |  |
| Half-life | day | 1.0/1.5 (default/ceiling) | ([6](#_ENREF_6)) |
|  |  |  |  |
| No. of infected birds | # | Site depended, 10,000 - 200,000 | USDA confidential data |
|  |  |  |  |
| % manure in dust | % | 5 (hen)  40 (turkey) | ([3](#_ENREF_3), [7](#_ENREF_7)) |
|  |  |  |  |
| Infectious dose | log EID_50_ | 3.5 (hen)  3 (turkey) | ([8](#_ENREF_8), [9](#_ENREF_9)) |
|  |  |  |  |
| Viral shedding rate | log EID_50_/[g feces] | 4/5 (default/ceiling) | ([10](#_ENREF_10), [11](#_ENREF_11)) |
|  |  |  |  |
| Viral survival @ 2 day | % | 60 (@24°C) | ([12](#_ENREF_12)) |
|  |  |  |  |
| Lung capacity | m^3^ | 1.4x10^-5^  7.7x10^-5^ | ([13](#_ENREF_13)) |
|  |  |  |  |
| Respiration rate | times/h | 1.6x10^3^ (hen)  2.4x10^3^ (turkey) | ([14](#_ENREF_14), [15](#_ENREF_15)) |
|  |  |  |  |
| Ventilation rate | m^3^/h/hen | 5 | ([16](#_ENREF_16)) |

**Table S2. Possible day (indicated by an ‘X’ in a box) of HPAI infection and origins of airborne virus for 77 Iowa cases during 2015 outbreak. Dates in parenthesis after origin states are the timeframe with infected cases.**

| **Outbreak**  **date** | **Type** | **Wisconsin (4/11/2015-5/6/2015)** | | | | | | | | | | | | | | | | | | | | |
| --- | --- | --- | --- | --- | --- | --- | --- | --- | --- | --- | --- | --- | --- | --- | --- | --- | --- | --- | --- | --- | --- | --- |
|  |  | d-1 | d-2 | d-3 | d-4 | d-5 | d-6 | d-7 | d-8 | d-9 | d-10 | d-11 | d-12 | d-13 | d-14 | d-15 | d-16 | d-17 | d-18 | d-19 | d-20 | d-21 |
| 4/12/15 | Turkey |  |  |  | **X** |  |  |  |  |  |  |  |  |  |  |  |  |  |  |  |  |  |
| 4/16/15 | Turkey |  |  |  |  |  |  |  |  | **X** |  |  |  |  |  |  |  |  |  |  |  |  |
| 4/19/15 | Layer |  |  |  |  |  |  |  |  |  |  |  |  |  |  |  |  |  |  |  |  |  |
| 4/24/15 | Layer |  |  |  |  |  |  |  |  |  |  |  |  |  |  |  |  |  |  |  |  |  |
| 4/24/15 | Layer |  |  |  |  |  |  |  |  |  |  |  |  |  |  |  |  |  |  |  |  |  |
| 4/25/15 | Turkey |  |  |  |  |  |  |  |  |  |  |  |  |  |  |  |  |  | **X** |  |  |  |
| 4/26/15 | Pullets |  |  |  |  |  |  |  |  |  |  |  |  |  |  |  |  |  |  |  |  |  |
| 4/26/15 | Layer |  |  |  |  |  |  |  |  |  |  |  |  |  |  |  |  |  |  |  |  |  |
| 4/27/15 | Turkey |  |  |  |  |  |  |  |  |  |  |  |  |  |  |  |  |  |  |  | **X** |  |
| 4/28/15 | Layer |  |  |  |  |  |  |  |  |  |  |  |  |  |  |  |  |  |  |  |  |  |
| 4/28/15 | Turkey |  |  |  |  |  |  |  |  |  |  |  |  |  |  |  |  |  |  |  |  |  |
| 4/28/15 | Breeder |  |  |  |  |  |  |  |  |  |  |  |  |  |  |  |  |  |  |  |  |  |
| 4/29/15 | Layer |  |  |  |  |  |  |  |  |  |  |  |  |  |  |  |  |  |  |  |  |  |
| 4/29/15 | Turkey |  |  |  |  |  |  |  |  |  |  |  |  |  |  |  |  |  |  |  |  |  |
| 4/29/15 | Layer |  |  |  |  |  |  |  |  |  |  |  |  |  |  |  |  |  |  |  |  |  |
| 4/29/15 | Turkey |  |  |  |  |  |  |  |  |  |  |  |  |  |  |  |  |  |  |  |  |  |
| 4/29/15 | Layer |  |  |  |  |  |  |  |  |  |  |  |  |  |  |  |  |  |  |  |  |  |
| 4/30/15 | Turkey |  |  |  |  |  |  |  |  |  |  |  |  |  |  |  |  |  |  |  |  |  |
| 4/30/15 | Layer |  |  |  |  | **X** |  |  |  |  |  |  |  |  |  |  |  |  |  |  |  |  |
| 4/30/15 | Turkey |  |  |  |  |  |  |  |  |  |  |  |  |  |  |  |  |  |  |  |  |  |
| 5/1/15 | Turkey |  |  |  |  |  |  |  |  |  |  |  |  |  |  |  |  |  |  |  |  |  |
| 5/1/15 | Backyard |  |  |  |  |  |  |  |  |  |  |  |  |  |  |  |  |  |  |  |  |  |
| 5/2/15 | Turkey |  |  |  |  |  |  |  |  |  |  |  |  |  |  |  |  |  |  |  |  |  |
| 5/2/15 | Layer |  |  |  |  |  |  |  |  |  |  |  |  |  |  |  |  |  |  |  |  |  |
| 5/3/15 | Turkey |  |  |  |  |  |  |  |  |  |  |  |  |  |  |  |  |  |  |  |  |  |
| 5/3/15 | Layer |  |  |  |  |  |  |  |  |  |  |  |  |  |  |  |  |  |  |  |  |  |
| 5/3/15 | Layer |  |  |  |  |  |  |  |  |  |  |  |  |  |  |  |  |  |  |  |  |  |
| 5/4/15 | Pullets |  |  |  |  |  |  |  |  |  |  |  |  |  |  |  |  |  |  |  |  |  |
| 5/4/15 | Layer |  |  |  |  |  |  |  |  |  |  |  |  |  |  |  |  |  |  |  |  |  |
| 5/5/15 | Turkey |  |  |  |  |  |  |  |  |  |  |  |  |  |  |  |  |  |  |  |  |  |
| 5/5/15 | Turkey |  |  |  |  |  |  |  |  |  |  |  |  |  |  |  |  |  |  |  |  |  |
| 5/5/15 | Pullets |  |  |  |  |  |  |  |  |  |  |  |  |  |  |  |  |  |  |  |  |  |
| 5/5/15 | Pullets |  |  |  |  |  |  |  |  |  |  |  |  |  |  |  |  |  |  |  |  |  |
| 5/5/15 | Pullets |  |  |  |  |  |  |  |  |  |  |  |  |  |  |  |  |  |  |  |  |  |
| 5/5/15 | Layer |  |  |  |  |  |  |  |  |  |  |  |  |  |  |  |  |  |  |  |  |  |
| 5/6/15 | Turkey |  |  |  |  |  |  |  |  |  |  |  |  |  |  |  |  |  |  |  |  |  |
| 5/6/15 | Pullets |  |  |  |  |  |  |  |  |  |  |  |  |  |  |  |  |  |  |  |  |  |
| 5/6/15 | Turkey |  |  |  |  |  |  |  |  |  |  |  |  |  |  |  |  |  |  |  |  |  |
| 5/6/15 | Turkey |  |  |  |  |  |  |  |  |  |  |  |  |  |  |  |  |  |  |  |  |  |
| 5/7/15 | Turkey |  |  |  |  |  |  |  |  |  |  |  |  |  |  |  |  |  |  |  |  |  |
| 5/7/15 | Turkey |  |  |  |  |  |  |  |  |  |  |  |  |  |  |  |  |  |  |  |  |  |
| 5/7/15 | Backyard |  |  |  |  |  |  |  |  |  |  |  |  |  |  |  |  |  |  |  |  |  |
| 5/7/15 | Layer |  |  |  |  |  |  |  |  |  |  |  |  |  |  |  |  |  |  |  |  |  |
| 5/8/15 | Turkey |  |  |  |  |  |  |  |  |  |  |  |  |  |  |  |  |  |  |  |  |  |
| 5/9/15 | Pullets |  |  |  | **X** |  |  |  |  |  |  |  |  |  |  |  |  |  |  |  |  |  |
| 5/10/15 | Turkey |  |  |  |  |  |  |  |  |  |  |  |  |  |  |  |  |  |  |  |  |  |
| 5/10/15 | Turkey |  |  |  |  |  |  |  |  |  |  |  |  |  |  |  |  |  |  |  |  |  |
| 5/11/15 | Pullets |  |  |  |  |  |  |  |  |  |  |  |  |  |  |  |  |  |  |  |  |  |
| 5/11/15 | Layer |  |  |  |  |  |  |  |  |  |  |  |  |  |  |  |  |  |  |  |  |  |
| 5/12/15 | Pullets |  |  |  |  |  |  |  |  |  |  |  |  |  |  |  |  |  |  |  |  |  |
| 5/12/15 | Layer |  |  |  |  |  |  |  |  |  |  |  |  |  |  |  |  |  |  |  |  |  |
| 5/12/15 | Pullets |  |  |  |  |  |  |  |  |  |  |  |  |  |  |  |  |  |  |  |  |  |
| 5/13/15 | Backyard |  |  |  |  |  |  |  |  |  |  |  |  |  |  |  |  |  |  |  |  |  |
| 5/14/15 | Backyard |  |  |  |  |  |  |  |  |  |  |  |  |  |  |  |  |  |  |  |  |  |
| 5/14/15 | Backyard |  |  |  |  |  |  |  |  |  |  |  |  |  |  |  |  |  |  |  |  |  |
| 5/15/15 | Turkey |  |  |  |  |  |  |  |  |  |  |  |  |  |  |  |  |  |  |  |  |  |
| 5/17/15 | Turkey |  |  |  |  |  |  |  |  |  |  |  |  |  |  |  |  |  |  |  |  |  |
| 5/18/15 | Pullets |  |  |  |  |  |  |  |  |  |  |  |  |  |  |  |  |  |  |  |  |  |
| 5/18/15 | Layer |  |  |  |  |  |  |  |  |  |  |  |  |  |  |  |  |  |  |  |  |  |
| 5/19/15 | Turkey |  |  |  |  |  |  |  |  |  |  |  |  |  |  |  |  |  |  |  |  |  |
| 5/19/15 | Turkey |  |  |  |  |  |  |  |  |  |  |  |  |  |  |  |  |  |  |  |  |  |
| 5/19/15 | Turkey |  |  |  |  |  |  |  |  |  |  |  |  |  |  |  |  |  |  |  |  |  |
| 5/20/15 | Turkey |  |  |  |  |  |  |  |  |  |  |  |  |  |  |  |  |  |  |  |  |  |
| 5/24/15 | Layer |  |  |  |  |  |  |  |  |  |  |  |  |  |  |  |  |  |  |  |  |  |
| 5/25/15 | Layer |  |  |  |  |  |  |  |  |  |  |  |  |  |  |  |  |  |  |  |  |  |
| 5/25/15 | Layer |  |  |  |  |  |  |  |  |  |  |  |  |  |  |  |  |  |  |  | **X** |  |
| 5/27/15 | Turkey |  |  |  |  |  |  |  |  |  |  |  |  |  |  |  |  |  |  |  |  |  |
| 5/27/15 | Pullets |  |  |  |  |  |  |  |  |  |  |  |  |  |  |  |  |  |  |  |  |  |
| 5/28/15 | Turkey |  |  |  |  |  |  |  |  |  |  |  |  |  |  |  |  |  |  |  |  |  |
| 5/29/15 | Turkey |  |  |  |  |  |  |  |  |  |  |  |  |  |  |  |  |  |  |  |  |  |
| 5/31/15 | Turkey |  |  |  |  |  |  |  |  |  |  |  |  |  |  |  |  |  |  |  |  |  |
| 6/1/15 | Pullets |  |  |  |  |  |  |  |  |  |  |  |  |  |  |  |  |  |  |  |  |  |
| 6/1/15 | Turkey |  |  |  |  |  |  |  |  |  |  |  |  |  |  |  |  |  |  |  |  |  |
| 6/2/15 | Turkey |  |  |  |  |  |  |  |  |  |  |  |  |  |  |  |  |  |  |  |  |  |
| 6/2/15 | Turkey |  |  |  |  |  |  |  |  |  |  |  |  |  |  |  |  |  |  |  |  |  |
| 6/4/15 | Hatchery |  |  |  |  |  |  |  |  |  |  |  |  |  |  |  |  |  |  |  |  |  |
| 6/15/15 | Layer |  |  |  |  |  |  |  |  |  |  |  |  |  |  |  |  |  |  |  |  |  |

| **Outbreak**  **date** | **Type** | **South Dakota (4/1/2015-6/1/2015)** | | | | | | | | | | | | | | | | | | | | |
| --- | --- | --- | --- | --- | --- | --- | --- | --- | --- | --- | --- | --- | --- | --- | --- | --- | --- | --- | --- | --- | --- | --- |
|  |  | d-1 | d-2 | d-3 | d-4 | d-5 | d-6 | d-7 | d-8 | d-9 | d-10 | d-11 | d-12 | d-13 | d-14 | d-15 | d-16 | d-17 | d-18 | d-19 | d-20 | d-21 |
| 4/12/15 | Turkey |  | **X** |  |  |  |  |  |  |  |  |  |  |  |  |  |  |  |  |  |  |  |
| 4/16/15 | Turkey |  |  |  |  |  | **X** |  |  |  |  |  |  |  |  |  |  |  |  |  |  |  |
| 4/19/15 | Layer |  |  |  | **X** | **X** | **X** |  |  |  |  |  |  |  |  |  | **X** | **X** |  |  |  |  |
| 4/24/15 | Layer |  | **X** | **X** | **X** | **X** |  |  |  |  |  |  |  |  | **X** |  |  |  |  |  |  | **X** |
| 4/24/15 | Layer |  |  |  |  | **X** |  |  |  |  |  |  |  |  | **X** |  |  |  |  |  |  | **X** |
| 4/25/15 | Turkey |  |  | **X** | **X** | **X** |  |  |  |  |  |  |  |  |  | **X** |  |  |  |  |  |  |
| 4/26/15 | Pullets |  |  |  |  | **X** | **X** |  |  |  |  |  |  |  |  |  |  |  |  |  |  |  |
| 4/26/15 | Layer |  |  |  |  | **X** |  | **X** |  |  |  |  |  |  |  |  |  | **X** |  |  |  |  |
| 4/27/15 | Turkey |  |  |  |  | **X** | **X** | **X** |  |  |  |  |  |  |  |  |  | **X** |  |  |  |  |
| 4/28/15 | Layer |  |  |  |  |  | **X** | **X** |  |  |  |  |  |  |  |  |  |  | **X** |  |  |  |
| 4/28/15 | Turkey |  |  |  |  |  |  | **X** |  |  |  |  |  |  |  |  |  |  |  | **X** |  |  |
| 4/28/15 | Breeder |  |  |  |  |  |  |  |  |  |  |  |  |  |  |  |  |  |  |  |  |  |
| 4/29/15 | Layer |  |  |  |  |  |  | **X** |  |  |  |  |  |  |  |  |  |  |  | **X** |  |  |
| 4/29/15 | Turkey |  |  |  |  |  |  | **X** |  |  |  |  |  |  |  |  |  |  |  | **X** |  |  |
| 4/29/15 | Layer |  |  |  |  |  |  |  | **X** |  |  |  |  |  |  |  |  | **X** |  | **X** |  |  |
| 4/29/15 | Turkey |  |  |  |  |  |  |  |  | **X** |  |  |  |  |  |  |  |  |  | **X** |  |  |
| 4/29/15 | Layer |  |  |  |  |  |  |  | **X** |  | **X** |  |  |  |  |  |  | **X** |  | **X** | **X** |  |
| 4/30/15 | Turkey |  |  |  |  |  |  |  |  |  |  |  |  |  |  |  |  |  |  |  | **X** |  |
| 4/30/15 | Layer |  |  |  |  |  |  |  |  |  | **X** |  |  |  |  |  |  |  |  |  |  |  |
| 4/30/15 | Turkey |  |  |  |  |  |  |  |  | **X** |  | **X** |  |  |  |  |  |  |  |  | **X** |  |
| 5/1/15 | Turkey |  |  |  |  |  |  |  |  |  |  | **X** | **X** |  |  |  |  |  | **X** | **X** |  | **X** |
| 5/1/15 | Backyard |  |  |  |  |  |  |  |  | **X** | **X** |  | **X** |  |  |  |  |  | **X** | **X** |  | **X** |
| 5/2/15 | Turkey |  |  |  |  |  |  |  |  |  | **X** | **X** | **X** |  |  |  |  |  |  |  |  |  |
| 5/2/15 | Layer |  |  |  |  |  |  |  |  |  |  | **X** |  | **X** |  |  |  |  |  | **X** | **X** |  |
| 5/3/15 | Turkey |  |  |  |  |  |  |  |  |  |  |  | **X** | **X** | **X** |  |  |  |  |  |  |  |
| 5/3/15 | Layer |  |  |  |  |  |  |  |  |  |  |  |  | **X** | **X** |  |  |  |  |  |  |  |
| 5/3/15 | Layer |  |  |  |  |  |  |  |  |  |  |  | **X** |  | **X** |  |  |  |  |  | **X** | **X** |
| 5/4/15 | Pullets |  |  |  |  |  |  |  |  |  |  |  |  | **X** |  | **X** |  |  |  |  |  | **X** |
| 5/4/15 | Layer |  |  |  |  |  |  |  |  |  |  |  |  | **X** | **X** | **X** |  |  |  |  |  | **X** |
| 5/5/15 | Turkey |  |  |  |  |  |  |  |  |  |  |  |  |  | **X** |  | **X** |  |  |  |  |  |
| 5/5/15 | Turkey |  |  |  |  |  |  |  |  |  |  |  |  |  | **X** | **X** |  |  |  |  |  |  |
| 5/5/15 | Pullets |  |  |  |  |  |  |  |  |  |  |  |  |  |  |  | **X** |  |  |  |  |  |
| 5/5/15 | Pullets |  |  |  |  |  |  |  |  |  |  |  |  |  | **X** |  | **X** |  |  |  |  |  |
| 5/5/15 | Pullets |  |  |  |  |  |  |  |  |  |  |  |  |  | **X** |  | **X** |  |  |  |  |  |
| 5/5/15 | Layer |  |  |  |  |  |  |  |  |  |  |  |  |  | **X** |  | **X** |  |  |  |  |  |
| 5/6/15 | Turkey |  |  |  |  |  |  |  |  |  |  |  |  |  |  | **X** | **X** |  |  |  |  |  |
| 5/6/15 | Pullets |  |  |  |  |  |  |  |  |  |  |  |  |  |  |  | **X** |  |  |  |  |  |
| 5/6/15 | Turkey |  |  |  |  |  |  |  |  |  |  |  |  |  |  | **X** | **X** |  | **X** |  |  |  |
| 5/6/15 | Turkey |  |  |  |  |  |  |  |  |  |  |  |  |  |  | **X** |  |  |  |  |  |  |
| 5/7/15 | Turkey |  |  |  |  |  |  |  |  |  |  |  |  |  |  |  | **X** |  | **X** | **X** |  |  |
| 5/7/15 | Turkey |  |  |  |  |  |  |  |  |  |  |  |  |  |  |  | **X** |  |  |  |  |  |
| 5/7/15 | Backyard |  |  |  |  |  |  |  |  |  |  |  |  |  |  |  |  | **X** | **X** |  |  |  |
| 5/7/15 | Layer |  |  |  |  |  |  |  |  |  |  |  |  |  |  |  | **X** | **X** | **X** |  |  |  |
| 5/8/15 | Turkey |  |  |  |  |  |  |  |  |  |  |  |  |  |  |  | **X** | **X** | **X** |  |  |  |
| 5/9/15 | Pullets |  |  |  |  |  |  |  |  |  |  |  |  |  |  |  |  |  |  |  |  |  |
| 5/10/15 | Turkey |  |  |  |  |  |  |  |  |  |  |  |  |  |  |  |  |  |  |  |  |  |
| 5/10/15 | Turkey |  |  |  |  |  |  |  |  |  |  |  |  |  |  |  |  |  | **X** |  | **X** |  |
| 5/11/15 | Pullets |  |  |  |  |  |  |  |  |  |  |  |  |  |  |  |  |  |  |  |  |  |
| 5/11/15 | Layer |  |  |  |  |  |  |  |  |  |  |  |  |  |  |  |  |  |  |  | **X** |  |
| 5/12/15 | Pullets |  |  |  |  |  |  |  |  |  |  |  |  |  |  |  |  |  |  |  |  | **X** |
| 5/12/15 | Layer |  |  |  |  |  |  |  |  |  |  |  |  |  |  |  |  |  |  |  |  | **X** |
| 5/12/15 | Pullets |  |  |  |  |  |  |  |  |  |  |  |  |  |  |  |  |  |  |  |  |  |
| 5/13/15 | Backyard |  |  |  |  |  |  |  |  |  |  |  |  |  |  |  |  |  |  |  |  |  |
| 5/14/15 | Backyard |  |  |  |  |  |  |  |  |  |  |  |  |  |  |  |  |  |  |  |  |  |
| 5/14/15 | Backyard |  |  |  |  |  |  |  |  |  |  |  |  |  |  |  |  |  |  |  |  |  |
| 5/15/15 | Turkey |  |  |  |  |  |  |  |  |  |  |  |  |  |  |  |  |  |  |  |  |  |
| 5/17/15 | Turkey |  |  |  |  |  |  |  |  |  |  |  |  |  |  |  |  |  |  |  |  |  |
| 5/18/15 | Pullets |  |  |  |  |  |  |  |  |  |  |  |  |  |  |  |  |  |  |  |  |  |
| 5/18/15 | Layer |  |  |  |  |  |  |  |  |  |  |  |  |  |  |  |  |  |  |  |  |  |
| 5/19/15 | Turkey | **X** |  |  |  |  |  |  |  |  |  |  |  |  |  |  |  |  |  |  |  |  |
| 5/19/15 | Turkey |  |  |  |  |  |  |  |  |  |  |  |  |  |  |  |  |  |  |  |  |  |
| 5/19/15 | Turkey |  |  |  |  |  |  |  |  |  |  |  |  |  |  |  |  |  |  |  |  |  |
| 5/20/15 | Turkey |  | **X** |  |  |  |  |  |  |  |  |  |  |  |  |  |  |  |  |  |  |  |
| 5/24/15 | Layer |  |  |  |  |  |  |  |  |  |  |  |  |  |  |  |  |  |  |  |  |  |
| 5/25/15 | Layer |  |  |  | **X** |  |  | **X** |  |  |  |  |  |  |  |  |  |  |  |  |  |  |
| 5/25/15 | Layer |  |  |  |  |  |  | **X** |  |  |  |  |  |  |  |  |  |  |  |  |  |  |
| 5/27/15 | Turkey |  |  |  |  |  |  |  |  | **X** |  |  |  |  |  |  |  |  |  |  |  |  |
| 5/27/15 | Pullets |  |  |  |  |  | **X** |  |  | **X** |  |  |  |  |  |  |  |  |  |  |  |  |
| 5/28/15 | Turkey |  |  |  |  |  |  |  |  |  |  |  |  |  |  |  |  |  |  |  |  |  |
| 5/29/15 | Turkey |  |  |  |  |  |  |  |  |  |  | **X** |  |  |  |  |  |  |  |  |  |  |
| 5/31/15 | Turkey |  |  |  |  |  |  |  |  |  |  |  |  | **X** |  |  |  |  |  |  |  |  |
| 6/1/15 | Pullets |  |  |  |  |  |  |  |  |  |  |  |  |  | **X** |  |  |  |  |  |  |  |
| 6/1/15 | Turkey |  |  |  |  |  |  |  |  |  |  |  |  |  | **X** |  |  |  |  |  |  |  |
| 6/2/15 | Turkey |  |  |  |  |  |  |  |  |  |  |  |  |  |  | **X** |  |  |  |  |  |  |
| 6/2/15 | Turkey |  |  |  |  |  |  |  |  |  |  |  |  |  |  | **X** |  |  |  |  |  |  |
| 6/4/15 | Hatchery |  |  |  |  |  | **X** |  |  | **X** |  |  |  |  |  | **X** | **X** |  |  |  |  |  |
| 6/15/15 | Layer |  |  |  |  |  |  | **X** |  |  |  |  |  |  |  |  |  |  |  |  |  |  |

| **Outbreak**  **date** | **Type** | **Minnesota (3/4/2015-6/5/2015)** | | | | | | | | | | | | | | | | | | | | |
| --- | --- | --- | --- | --- | --- | --- | --- | --- | --- | --- | --- | --- | --- | --- | --- | --- | --- | --- | --- | --- | --- | --- |
|  |  | d-1 | d-2 | d-3 | d-4 | d-5 | d-6 | d-7 | d-8 | d-9 | d-10 | d-11 | d-12 | d-13 | d-14 | d-15 | d-16 | d-17 | d-18 | d-19 | d-20 | d-21 |
| 4/12/15 | Turkey |  |  | **X** |  |  |  |  | **X** |  | **X** |  |  |  |  |  |  | **X** |  |  |  |  |
| 4/16/15 | Turkey |  |  |  |  |  |  | **X** |  |  |  |  |  | **X** |  |  |  |  |  |  |  | **X** |
| 4/19/15 | Layer |  |  |  |  |  |  |  |  |  | **X** | **X** |  |  |  |  | **X** | **X** |  |  |  |  |
| 4/24/15 | Layer |  |  |  |  |  |  |  |  |  |  |  |  |  |  | **X** | **X** |  |  |  |  |  |
| 4/24/15 | Layer |  |  |  |  |  |  |  |  |  |  |  |  |  |  | **X** | **X** |  |  |  |  |  |
| 4/25/15 | Turkey |  |  |  | **X** |  |  |  |  |  |  |  |  |  |  |  | **X** |  |  |  |  |  |
| 4/26/15 | Pullets |  |  |  | **X** | **X** |  |  |  |  |  |  |  |  |  |  |  | **X** | **X** |  |  |  |
| 4/26/15 | Layer |  |  |  |  |  |  |  |  |  |  |  |  |  |  |  | **X** | **X** |  | **X** |  |  |
| 4/27/15 | Turkey |  |  |  |  |  |  |  |  |  |  |  |  |  |  |  |  |  | **X** |  |  |  |
| 4/28/15 | Layer |  |  |  |  |  | **X** | **X** |  |  |  |  |  |  |  |  |  |  |  |  | **X** |  |
| 4/28/15 | Turkey |  |  |  |  |  |  |  |  |  |  |  |  |  |  |  |  |  |  |  | **X** |  |
| 4/28/15 | Breeder |  |  |  |  |  | **X** | **X** | **X** | **X** |  |  |  |  |  |  |  |  |  | **X** | **X** |  |
| 4/29/15 | Layer | **X** |  |  |  |  |  |  |  | **X** |  |  |  |  |  |  |  |  |  |  | **X** |  |
| 4/29/15 | Turkey |  |  |  |  |  |  | **X** |  |  |  |  |  |  |  |  |  |  |  |  | **X** |  |
| 4/29/15 | Layer |  |  |  |  |  |  |  |  |  |  |  |  |  |  |  |  |  |  |  | **X** |  |
| 4/29/15 | Turkey |  |  |  |  |  |  |  | **X** |  |  |  |  |  |  |  |  |  |  |  | **X** |  |
| 4/29/15 | Layer | **X** |  |  |  |  |  |  |  |  |  |  |  |  |  |  |  |  |  |  | **X** | **X** |
| 4/30/15 | Turkey |  |  |  |  |  |  |  | **X** | **X** |  | **X** |  |  |  |  |  |  |  |  |  | **X** |
| 4/30/15 | Layer |  |  |  |  |  |  |  |  |  |  |  |  |  |  |  |  |  |  |  |  | **X** |
| 4/30/15 | Turkey |  |  |  |  |  |  |  |  |  |  |  |  |  |  |  |  |  |  |  |  | **X** |
| 5/1/15 | Turkey |  | **X** |  |  |  |  |  |  |  |  |  |  |  |  |  |  |  |  |  |  |  |
| 5/1/15 | Backyard |  | **X** | **X** |  |  |  |  |  |  |  |  |  |  | **X** |  |  |  |  |  |  |  |
| 5/2/15 | Turkey |  |  | **X** |  |  |  |  |  |  | **X** | **X** |  |  |  | **X** |  |  |  |  |  |  |
| 5/2/15 | Layer |  |  | **X** | **X** |  |  |  |  |  |  |  |  |  |  |  |  |  |  |  |  |  |
| 5/3/15 | Turkey |  |  |  |  |  |  |  |  |  |  |  |  |  |  |  |  |  |  |  |  |  |
| 5/3/15 | Layer |  |  |  | **X** | **X** |  |  |  |  |  |  |  |  |  |  |  |  |  |  |  |  |
| 5/3/15 | Layer |  |  |  | **X** | **X** |  |  |  |  |  |  |  |  |  |  |  |  |  |  |  |  |
| 5/4/15 | Pullets | **X** |  |  |  | **X** | **X** |  |  |  |  |  |  |  |  |  |  |  |  |  |  |  |
| 5/4/15 | Layer |  |  |  |  | **X** |  | **X** |  |  |  |  | **X** | **X** |  | **X** |  |  |  |  |  |  |
| 5/5/15 | Turkey |  |  |  |  |  |  |  |  |  |  |  |  |  |  |  |  |  |  |  |  |  |
| 5/5/15 | Turkey |  |  |  |  |  |  |  |  |  |  |  |  | **X** | **X** |  |  |  |  |  |  |  |
| 5/5/15 | Pullets |  | **X** |  |  |  | **X** |  |  |  |  |  |  |  |  |  |  |  |  |  |  |  |
| 5/5/15 | Pullets |  | **X** |  |  |  | **X** |  |  |  |  |  |  |  |  |  |  |  |  |  |  |  |
| 5/5/15 | Pullets |  | **X** |  |  |  | **X** |  |  |  |  |  |  |  |  |  |  |  |  |  |  |  |
| 5/5/15 | Layer |  | **X** |  |  |  | **X** | **X** | **X** |  |  |  |  |  |  |  |  |  |  |  |  |  |
| 5/6/15 | Turkey |  | **X** |  |  |  |  |  |  |  |  |  |  |  |  |  |  |  |  |  |  |  |
| 5/6/15 | Pullets |  | **X** | **X** |  |  |  | **X** |  |  |  |  |  |  | **X** | **X** |  |  |  |  |  |  |
| 5/6/15 | Turkey |  |  |  |  |  |  |  |  |  |  |  |  |  | **X** | **X** |  |  |  | **X** |  |  |
| 5/6/15 | Turkey |  |  |  |  |  |  |  |  |  |  |  |  |  |  |  |  |  |  |  |  |  |
| 5/7/15 | Turkey |  |  |  |  |  |  |  | **X** |  |  |  |  |  |  |  |  |  |  |  |  |  |
| 5/7/15 | Turkey |  |  |  |  |  |  |  | **X** | **X** |  |  |  |  |  |  |  |  |  |  |  |  |
| 5/7/15 | Backyard |  |  | **X** | **X** |  |  |  | **X** |  |  |  |  |  |  | **X** | **X** |  |  |  | **X** |  |
| 5/7/15 | Layer |  |  |  |  |  |  |  | **X** |  | **X** |  |  |  |  | **X** | **X** |  | **X** |  |  |  |
| 5/8/15 | Turkey |  |  |  |  |  |  |  |  |  |  |  |  |  |  |  |  | **X** |  |  |  |  |
| 5/9/15 | Pullets |  |  |  |  |  |  |  |  |  |  |  |  |  |  |  | **X** | **X** |  | **X** |  |  |
| 5/10/15 | Turkey |  | **X** |  |  |  |  |  |  |  |  |  |  |  |  |  |  |  |  |  |  |  |
| 5/10/15 | Turkey |  | **X** |  |  |  |  |  |  |  | **X** |  |  |  |  |  |  |  |  |  |  |  |
| 5/11/15 | Pullets |  | **X** | **X** |  |  |  |  |  |  |  |  | **X** | **X** |  |  |  |  |  | **X** |  |  |
| 5/11/15 | Layer | **X** | **X** | **X** |  |  |  | **X** | **X** |  |  |  | **X** | **X** | **X** |  |  |  |  |  |  |  |
| 5/12/15 | Pullets |  | **X** | **X** | **X** |  |  |  |  | **X** | **X** |  |  | **X** |  |  |  |  |  |  |  |  |
| 5/12/15 | Layer |  | **X** | **X** | **X** |  |  |  | **X** | **X** |  |  |  | **X** | **X** |  |  |  |  |  |  |  |
| 5/12/15 | Pullets |  | **X** | **X** | **X** |  |  |  | **X** | **X** |  |  |  | **X** | **X** |  |  |  |  |  |  |  |
| 5/13/15 | Backyard |  |  | **X** | **X** | **X** |  |  |  |  |  |  |  |  | **X** |  |  |  |  |  |  |  |
| 5/14/15 | Backyard |  |  |  | **X** | **X** | **X** |  |  |  |  |  |  |  |  | **X** |  |  |  |  |  |  |
| 5/14/15 | Backyard |  |  |  | **X** | **X** | **X** |  |  |  |  |  |  |  |  | **X** |  |  |  |  |  |  |
| 5/15/15 | Turkey |  |  |  |  |  |  | **X** |  |  |  |  |  |  |  |  |  |  |  |  |  |  |
| 5/17/15 | Turkey |  |  |  |  |  |  |  | **X** |  |  |  |  |  |  |  |  |  | **X** | **X** |  |  |
| 5/18/15 | Pullets |  |  |  |  |  |  |  |  |  | **X** |  |  |  |  |  | **X** |  |  | **X** | **X** |  |
| 5/18/15 | Layer |  |  |  |  |  |  |  | **X** | **X** | **X** |  |  |  | **X** | **X** |  |  |  | **X** | **X** |  |
| 5/19/15 | Turkey |  |  |  |  |  |  |  |  |  |  |  |  |  |  |  |  |  |  |  |  |  |
| 5/19/15 | Turkey |  |  |  |  |  |  |  |  |  |  | **X** |  |  |  |  |  |  |  |  |  |  |
| 5/19/15 | Turkey |  |  |  |  |  |  |  |  |  |  | **X** |  |  |  |  |  |  |  |  |  |  |
| 5/20/15 | Turkey |  |  |  |  |  |  |  |  |  |  |  | **X** |  |  |  |  |  |  |  |  |  |
| 5/24/15 | Layer |  |  |  |  |  |  |  |  |  |  |  |  |  |  |  |  |  |  |  |  |  |
| 5/25/15 | Layer |  |  |  |  |  | **X** |  |  |  |  |  |  |  | **X** |  |  |  |  |  |  |  |
| 5/25/15 | Layer |  |  |  |  |  |  |  |  |  |  |  |  |  | **X** |  |  | **X** |  |  |  |  |
| 5/27/15 | Turkey | **X** |  |  |  |  |  |  | **X** |  |  |  |  |  |  | **X** |  |  |  |  |  |  |
| 5/27/15 | Pullets | **X** |  |  |  |  |  |  | **X** |  |  |  |  |  |  |  |  |  |  | **X** |  |  |
| 5/28/15 | Turkey |  |  |  |  |  |  |  |  | **X** |  |  |  |  |  |  |  |  |  |  |  |  |
| 5/29/15 | Turkey |  |  | **X** |  |  |  |  |  |  |  |  |  |  |  |  |  | **X** |  |  |  |  |
| 5/31/15 | Turkey | **X** | **X** |  |  | **X** |  |  |  |  |  |  | **X** |  |  |  |  |  |  | **X** |  |  |
| 6/1/15 | Pullets |  | **X** |  |  |  |  |  |  |  |  |  |  | **X** |  |  |  |  |  |  | **X** |  |
| 6/1/15 | Turkey |  |  |  |  |  | **X** |  |  |  |  |  |  | **X** |  |  |  |  |  |  |  |  |
| 6/2/15 | Turkey |  |  |  |  |  |  | **X** |  |  |  |  |  |  | **X** |  |  |  |  |  | **X** | **X** |
| 6/2/15 | Turkey |  |  | **X** |  |  |  | **X** |  |  |  |  |  |  | **X** |  |  |  |  |  |  |  |
| 6/4/15 | Hatchery |  |  |  |  |  |  |  |  | **X** |  |  |  |  |  |  |  |  |  |  |  |  |
| 6/15/15 | Layer |  |  |  |  |  |  |  |  |  |  |  |  |  |  |  |  | **X** |  |  | **X** |  |

| **Outbreak**  **date** | **Type** | **Missouri (3/9/2015-5/10/2015)** | | | | | | | | | | | | | | | | | | | | |
| --- | --- | --- | --- | --- | --- | --- | --- | --- | --- | --- | --- | --- | --- | --- | --- | --- | --- | --- | --- | --- | --- | --- |
|  |  | d-1 | d-2 | d-3 | d-4 | d-5 | d-6 | d-7 | d-8 | d-9 | d-10 | d-11 | d-12 | d-13 | d-14 | d-15 | d-16 | d-17 | d-18 | d-19 | d-20 | d-21 |
| 4/12/15 | Turkey |  |  |  |  |  |  |  |  |  |  |  |  |  |  |  |  |  |  |  |  |  |
| 4/16/15 | Turkey |  |  |  |  |  |  |  |  |  |  |  |  |  |  |  |  |  |  |  |  |  |
| 4/19/15 | Layer |  |  |  |  |  |  |  |  |  |  |  |  |  |  |  |  |  |  |  |  |  |
| 4/24/15 | Layer |  |  |  |  |  |  |  |  |  |  |  |  |  |  |  |  |  |  |  |  |  |
| 4/24/15 | Layer |  |  |  |  |  |  |  |  |  |  |  |  |  |  |  |  |  |  |  |  |  |
| 4/25/15 | Turkey |  |  |  |  |  |  |  |  |  |  |  |  |  |  |  |  |  |  |  |  |  |
| 4/26/15 | Pullets |  |  |  |  |  |  |  |  |  |  |  |  |  |  |  |  |  |  |  |  |  |
| 4/26/15 | Layer |  |  |  |  |  |  |  |  |  |  |  |  |  |  |  |  |  |  |  |  |  |
| 4/27/15 | Turkey |  |  |  |  |  |  |  |  |  |  |  |  |  |  |  |  |  |  |  |  |  |
| 4/28/15 | Layer |  |  |  |  |  |  |  |  |  |  |  |  |  |  |  |  |  |  |  |  |  |
| 4/28/15 | Turkey |  |  |  |  |  |  |  |  |  |  |  |  |  |  |  |  |  |  |  |  |  |
| 4/28/15 | Breeder |  |  |  |  |  |  |  |  |  |  |  |  |  |  |  |  |  |  |  |  |  |
| 4/29/15 | Layer |  |  |  |  |  |  |  |  |  |  |  |  |  |  |  |  |  |  |  |  |  |
| 4/29/15 | Turkey |  |  |  |  |  |  |  |  |  |  |  |  |  |  |  |  |  |  |  |  |  |
| 4/29/15 | Layer |  |  |  |  |  |  |  |  |  |  |  |  |  |  |  |  |  |  |  |  |  |
| 4/29/15 | Turkey |  |  |  |  |  |  |  |  |  |  |  |  |  |  |  |  |  |  |  |  |  |
| 4/29/15 | Layer |  |  |  |  |  |  |  |  |  |  |  |  |  |  |  |  |  |  |  |  |  |
| 4/30/15 | Turkey |  |  |  |  |  |  |  |  |  |  |  |  |  |  |  |  |  |  |  |  |  |
| 4/30/15 | Layer |  |  |  |  |  |  |  |  |  |  |  |  |  |  |  |  |  |  |  |  |  |
| 4/30/15 | Turkey |  |  |  |  |  |  |  |  |  |  |  |  |  |  |  |  |  |  |  |  |  |
| 5/1/15 | Turkey |  |  |  |  |  |  |  |  |  |  |  |  |  |  |  |  |  |  |  |  |  |
| 5/1/15 | Backyard |  |  |  |  |  |  |  |  |  |  |  |  |  |  |  |  |  |  |  |  |  |
| 5/2/15 | Turkey |  |  |  |  |  |  |  |  |  |  |  |  |  |  |  |  |  |  |  |  |  |
| 5/2/15 | Layer |  |  |  |  |  |  |  |  |  |  |  |  |  |  |  |  |  |  |  |  |  |
| 5/3/15 | Turkey |  |  |  |  |  |  |  |  |  |  |  |  |  |  |  |  |  |  |  |  |  |
| 5/3/15 | Layer |  |  |  |  |  |  |  |  |  |  |  |  |  |  |  |  |  |  |  |  |  |
| 5/3/15 | Layer |  |  |  |  |  |  |  |  |  |  |  |  |  |  |  |  |  |  |  |  |  |
| 5/4/15 | Pullets |  |  |  |  |  |  |  |  |  |  |  |  |  |  |  |  |  |  |  |  |  |
| 5/4/15 | Layer |  |  |  |  |  |  |  |  |  |  |  |  |  |  |  |  |  |  |  |  |  |
| 5/5/15 | Turkey |  |  |  |  |  |  |  |  |  |  |  |  |  |  |  |  |  |  |  |  |  |
| 5/5/15 | Turkey |  |  |  |  |  |  |  |  |  |  |  |  |  |  |  |  |  |  |  |  |  |
| 5/5/15 | Pullets |  |  |  |  |  |  |  |  |  |  |  |  |  |  |  |  |  |  |  |  |  |
| 5/5/15 | Pullets |  |  |  |  |  |  |  |  |  |  |  |  |  |  |  |  |  |  |  |  |  |
| 5/5/15 | Pullets |  |  |  |  |  |  |  |  |  |  |  |  |  |  |  |  |  |  |  |  |  |
| 5/5/15 | Layer |  |  |  |  |  |  |  |  |  |  |  |  |  |  |  |  |  |  |  |  |  |
| 5/6/15 | Turkey |  |  |  |  |  |  |  |  |  |  |  |  |  |  |  |  |  |  |  |  |  |
| 5/6/15 | Pullets |  |  |  |  |  |  |  |  |  |  |  |  |  |  |  |  |  |  |  |  |  |
| 5/6/15 | Turkey |  |  |  |  |  |  |  |  |  |  |  |  |  |  |  |  |  |  |  |  |  |
| 5/6/15 | Turkey |  |  |  |  |  |  |  |  |  |  |  |  |  |  |  |  |  |  |  |  |  |
| 5/7/15 | Turkey |  |  |  |  |  |  |  |  |  |  |  |  |  |  |  |  |  |  |  |  |  |
| 5/7/15 | Turkey |  |  |  |  |  |  |  |  |  |  |  |  |  |  |  |  |  |  |  |  |  |
| 5/7/15 | Backyard |  |  |  |  |  |  |  |  |  |  |  |  |  |  |  |  |  |  |  |  |  |
| 5/7/15 | Layer |  |  |  |  |  |  |  |  |  |  |  |  |  |  |  |  |  |  |  |  |  |
| 5/8/15 | Turkey |  |  |  |  |  |  |  |  |  |  |  |  |  |  |  |  |  |  |  |  |  |
| 5/9/15 | Pullets |  |  |  |  |  |  |  |  |  |  |  |  |  |  |  |  |  |  |  |  |  |
| 5/10/15 | Turkey |  |  |  |  |  |  |  |  |  |  |  |  |  |  |  |  |  |  |  |  |  |
| 5/10/15 | Turkey |  |  |  |  |  |  |  |  |  |  |  |  |  |  |  |  |  |  |  |  |  |
| 5/11/15 | Pullets |  |  |  |  |  |  |  |  |  |  |  |  |  |  |  |  |  |  |  |  |  |
| 5/11/15 | Layer |  |  |  |  |  |  |  |  |  |  |  |  |  |  |  |  |  |  |  |  |  |
| 5/12/15 | Pullets |  |  |  |  |  |  |  |  |  |  |  |  |  |  |  |  |  |  |  |  |  |
| 5/12/15 | Layer |  |  |  |  |  |  |  |  |  |  |  |  |  |  |  |  |  |  |  |  |  |
| 5/12/15 | Pullets |  |  |  |  |  |  |  |  |  |  |  |  |  |  |  |  |  |  |  |  |  |
| 5/13/15 | Backyard |  |  |  |  |  |  |  |  |  |  |  |  |  |  |  |  |  |  |  |  |  |
| 5/14/15 | Backyard |  |  |  |  |  |  |  |  |  |  |  |  |  |  |  |  |  |  |  |  |  |
| 5/14/15 | Backyard |  |  |  |  |  |  |  |  |  |  |  |  |  |  |  |  |  |  |  |  |  |
| 5/15/15 | Turkey |  | **X** |  |  |  |  |  |  |  |  |  |  |  |  |  |  |  |  |  |  |  |
| 5/17/15 | Turkey |  |  |  |  |  |  |  |  |  |  |  |  |  |  |  |  |  |  |  |  |  |
| 5/18/15 | Pullets |  |  |  |  |  |  |  |  |  |  |  |  |  |  |  |  |  |  |  |  |  |
| 5/18/15 | Layer |  |  |  |  |  |  |  |  |  |  |  |  |  |  |  |  |  |  |  |  |  |
| 5/19/15 | Turkey |  |  |  |  | **X** |  |  |  |  |  |  |  |  |  |  |  |  |  |  |  |  |
| 5/19/15 | Turkey |  |  |  |  |  |  |  |  |  |  |  |  |  |  |  |  |  |  |  |  |  |
| 5/19/15 | Turkey |  |  |  |  |  |  |  |  |  |  |  |  |  |  |  |  |  |  |  |  |  |
| 5/20/15 | Turkey |  |  |  |  |  | **X** |  |  |  |  |  |  |  |  |  |  |  |  |  |  |  |
| 5/24/15 | Layer |  |  |  |  |  |  |  |  |  |  |  |  |  |  |  |  |  |  |  |  |  |
| 5/25/15 | Layer |  |  |  |  |  |  |  |  |  |  |  |  |  |  |  | **X** |  |  |  |  |  |
| 5/25/15 | Layer | **X** |  |  |  |  | **X** |  |  |  |  |  |  | **X** |  |  |  |  |  |  |  |  |
| 5/27/15 | Turkey |  |  | **X** |  |  |  |  |  |  |  |  |  |  |  |  |  |  | **X** |  |  |  |
| 5/27/15 | Pullets |  |  | **X** |  |  |  |  |  |  |  |  | **X** |  |  |  |  |  |  |  |  |  |
| 5/28/15 | Turkey |  |  |  |  |  |  |  |  |  |  |  |  |  |  |  |  |  |  |  |  |  |
| 5/29/15 | Turkey |  |  |  |  | **X** |  |  |  |  |  |  |  |  |  |  | **X** |  |  |  |  |  |
| 5/31/15 | Turkey |  |  |  |  |  |  |  |  |  |  |  |  |  |  |  |  | **X** |  |  |  |  |
| 6/1/15 | Pullets |  |  |  |  |  |  |  |  |  |  |  |  |  |  |  |  |  |  |  |  |  |
| 6/1/15 | Turkey |  |  |  |  |  |  |  | **X** |  |  |  |  |  |  |  |  |  |  |  |  |  |
| 6/2/15 | Turkey |  |  |  |  |  |  |  |  | **X** |  |  |  |  |  |  |  |  |  | **X** |  |  |
| 6/2/15 | Turkey |  |  |  |  |  |  |  |  |  |  |  |  |  |  |  |  |  |  |  |  |  |
| 6/4/15 | Hatchery |  |  |  |  |  |  |  |  |  |  |  |  |  |  |  |  |  |  |  |  |  |
| 6/15/15 | Layer |  |  |  |  |  |  |  |  |  |  |  |  |  |  |  |  |  |  |  |  |  |

| **Outbreak**  **date** | **Type** | **Nebraska (5/12/2015-6/4/2015)** | | | | | | | | | | | | | | | | | | | | |
| --- | --- | --- | --- | --- | --- | --- | --- | --- | --- | --- | --- | --- | --- | --- | --- | --- | --- | --- | --- | --- | --- | --- |
|  |  | d-1 | d-2 | d-3 | d-4 | d-5 | d-6 | d-7 | d-8 | d-9 | d-10 | d-11 | d-12 | d-13 | d-14 | d-15 | d-16 | d-17 | d-18 | d-19 | d-20 | d-21 |
| 4/12/15 | Turkey |  |  |  |  |  |  |  |  |  |  |  |  |  |  |  |  |  |  |  |  |  |
| 4/16/15 | Turkey |  |  |  |  |  |  |  |  |  |  |  |  |  |  |  |  |  |  |  |  |  |
| 4/19/15 | Layer |  |  |  |  |  |  |  |  |  |  |  |  |  |  |  |  |  |  |  |  |  |
| 4/24/15 | Layer |  |  |  |  |  |  |  |  |  |  |  |  |  |  |  |  |  |  |  |  |  |
| 4/24/15 | Layer |  |  |  |  |  |  |  |  |  |  |  |  |  |  |  |  |  |  |  |  |  |
| 4/25/15 | Turkey |  |  |  |  |  |  |  |  |  |  |  |  |  |  |  |  |  |  |  |  |  |
| 4/26/15 | Pullets |  |  |  |  |  |  |  |  |  |  |  |  |  |  |  |  |  |  |  |  |  |
| 4/26/15 | Layer |  |  |  |  |  |  |  |  |  |  |  |  |  |  |  |  |  |  |  |  |  |
| 4/27/15 | Turkey |  |  |  |  |  |  |  |  |  |  |  |  |  |  |  |  |  |  |  |  |  |
| 4/28/15 | Layer |  |  |  |  |  |  |  |  |  |  |  |  |  |  |  |  |  |  |  |  |  |
| 4/28/15 | Turkey |  |  |  |  |  |  |  |  |  |  |  |  |  |  |  |  |  |  |  |  |  |
| 4/28/15 | Breeder |  |  |  |  |  |  |  |  |  |  |  |  |  |  |  |  |  |  |  |  |  |
| 4/29/15 | Layer |  |  |  |  |  |  |  |  |  |  |  |  |  |  |  |  |  |  |  |  |  |
| 4/29/15 | Turkey |  |  |  |  |  |  |  |  |  |  |  |  |  |  |  |  |  |  |  |  |  |
| 4/29/15 | Layer |  |  |  |  |  |  |  |  |  |  |  |  |  |  |  |  |  |  |  |  |  |
| 4/29/15 | Turkey |  |  |  |  |  |  |  |  |  |  |  |  |  |  |  |  |  |  |  |  |  |
| 4/29/15 | Layer |  |  |  |  |  |  |  |  |  |  |  |  |  |  |  |  |  |  |  |  |  |
| 4/30/15 | Turkey |  |  |  |  |  |  |  |  |  |  |  |  |  |  |  |  |  |  |  |  |  |
| 4/30/15 | Layer |  |  |  |  |  |  |  |  |  |  |  |  |  |  |  |  |  |  |  |  |  |
| 4/30/15 | Turkey |  |  |  |  |  |  |  |  |  |  |  |  |  |  |  |  |  | **X** |  |  |  |
| 5/1/15 | Turkey |  |  |  |  |  |  |  |  |  |  |  |  |  |  |  |  |  |  |  |  |  |
| 5/1/15 | Backyard |  |  |  |  |  |  |  |  |  |  |  |  |  |  |  |  |  |  |  |  |  |
| 5/2/15 | Turkey |  |  |  |  |  |  |  |  |  |  |  |  |  |  |  |  |  |  |  |  |  |
| 5/2/15 | Layer |  |  |  |  |  |  |  |  |  |  |  |  |  |  |  |  |  |  |  |  |  |
| 5/3/15 | Turkey |  |  |  |  |  |  |  |  |  |  |  |  |  |  |  |  |  |  |  |  |  |
| 5/3/15 | Layer |  |  |  |  |  |  |  |  |  |  |  |  |  |  |  |  |  |  |  |  |  |
| 5/3/15 | Layer |  |  |  |  |  |  |  |  |  |  |  |  |  |  |  |  |  |  |  |  |  |
| 5/4/15 | Pullets |  |  |  |  |  |  |  |  |  |  |  |  |  |  |  |  |  |  |  |  |  |
| 5/4/15 | Layer |  |  |  |  |  |  |  |  |  |  |  |  |  |  |  |  |  |  |  |  |  |
| 5/5/15 | Turkey |  |  |  |  |  |  |  |  |  |  |  |  |  |  |  |  |  |  |  |  |  |
| 5/5/15 | Turkey |  |  |  |  |  |  |  |  |  |  |  |  |  |  |  |  |  |  |  |  |  |
| 5/5/15 | Pullets |  |  |  |  |  |  |  |  |  |  |  |  |  |  |  |  |  |  |  |  |  |
| 5/5/15 | Pullets |  |  |  |  |  |  |  |  |  |  |  |  |  |  |  |  |  |  |  |  |  |
| 5/5/15 | Pullets |  |  |  |  |  |  |  |  |  |  |  |  |  |  |  |  |  |  |  |  |  |
| 5/5/15 | Layer |  |  |  |  |  |  |  |  |  |  |  |  |  |  |  |  |  |  |  |  |  |
| 5/6/15 | Turkey |  |  |  |  |  |  |  |  |  |  |  |  |  |  |  |  |  |  |  |  |  |
| 5/6/15 | Pullets |  |  |  |  |  |  |  |  |  |  |  |  |  |  |  |  |  |  |  |  |  |
| 5/6/15 | Turkey |  |  |  |  |  |  |  |  |  |  |  |  |  |  |  |  |  |  |  |  |  |
| 5/6/15 | Turkey |  |  |  |  |  |  |  |  |  |  |  |  |  |  |  |  |  |  |  |  |  |
| 5/7/15 | Turkey |  |  |  |  |  |  |  |  |  |  |  |  |  |  |  |  |  |  |  |  |  |
| 5/7/15 | Turkey |  |  |  |  |  |  |  |  |  |  |  |  |  |  |  |  |  |  |  |  |  |
| 5/7/15 | Backyard |  |  |  |  |  |  |  |  |  |  |  |  |  |  |  |  |  |  |  |  |  |
| 5/7/15 | Layer |  |  |  |  |  |  |  |  |  |  |  |  |  |  |  |  |  |  |  |  |  |
| 5/8/15 | Turkey |  |  |  |  |  |  |  |  |  |  |  |  |  |  |  |  |  |  |  |  |  |
| 5/9/15 | Pullets |  |  |  |  |  |  |  |  |  |  |  |  |  |  |  |  |  |  |  |  |  |
| 5/10/15 | Turkey |  |  |  |  |  |  |  |  |  |  |  |  |  |  |  |  |  |  |  |  |  |
| 5/10/15 | Turkey |  |  |  |  |  |  |  |  |  |  |  |  |  |  |  |  |  |  |  |  |  |
| 5/11/15 | Pullets |  |  |  |  |  |  |  |  |  |  |  |  |  |  |  |  |  |  |  |  |  |
| 5/11/15 | Layer |  |  |  |  |  |  |  |  |  |  |  |  |  |  |  |  |  |  |  |  |  |
| 5/12/15 | Pullets |  |  |  |  |  |  |  |  |  |  |  |  |  |  |  |  |  |  |  |  |  |
| 5/12/15 | Layer |  |  |  |  |  |  |  |  |  |  |  |  |  |  |  |  |  |  |  |  |  |
| 5/12/15 | Pullets |  |  |  |  |  |  |  |  |  |  |  |  |  |  |  |  |  |  |  |  |  |
| 5/13/15 | Backyard |  |  |  |  |  |  |  |  |  |  |  |  |  |  |  |  |  |  |  |  |  |
| 5/14/15 | Backyard |  |  |  |  |  |  |  |  |  |  |  |  |  |  |  |  |  |  |  |  |  |
| 5/14/15 | Backyard |  |  |  |  |  |  |  |  |  |  |  |  |  |  |  |  |  |  |  |  |  |
| 5/15/15 | Turkey |  |  |  |  |  |  |  |  |  |  |  |  |  |  |  |  |  |  |  |  |  |
| 5/17/15 | Turkey |  |  |  |  |  |  |  |  |  |  |  |  |  |  |  |  |  |  |  |  |  |
| 5/18/15 | Pullets | **X** |  |  |  |  |  |  |  |  |  |  |  |  |  |  |  |  |  |  |  |  |
| 5/18/15 | Layer | **X** |  |  |  |  |  |  |  |  |  |  |  |  |  |  |  |  |  |  |  |  |
| 5/19/15 | Turkey |  | **X** |  |  |  |  |  | **X** |  |  |  |  |  |  |  |  |  |  |  |  |  |
| 5/19/15 | Turkey | **X** |  |  |  |  |  |  | **X** |  |  |  |  |  |  |  |  |  |  |  |  |  |
| 5/19/15 | Turkey |  | **X** |  |  |  |  |  | **X** |  |  |  |  |  |  |  |  |  |  |  |  |  |
| 5/20/15 | Turkey |  |  | **X** |  |  |  |  |  | **X** |  |  |  |  |  |  |  |  |  |  |  |  |
| 5/24/15 | Layer |  |  |  |  |  |  |  |  |  |  |  |  |  |  |  |  |  |  |  |  |  |
| 5/25/15 | Layer |  |  |  |  |  |  |  |  |  |  |  |  |  |  | **X** |  |  |  |  |  |  |
| 5/25/15 | Layer |  |  |  |  |  |  |  | **X** |  |  |  |  |  |  |  |  |  |  |  |  |  |
| 5/27/15 | Turkey |  |  |  |  |  |  |  |  |  |  |  |  |  |  |  | **X** |  |  |  |  |  |
| 5/27/15 | Pullets |  |  |  |  |  |  |  |  |  | **X** |  |  |  |  |  |  |  |  |  |  |  |
| 5/28/15 | Turkey |  |  |  |  |  |  |  |  |  |  | **X** |  |  |  |  |  | **X** |  |  |  |  |
| 5/29/15 | Turkey |  |  |  |  |  |  |  |  |  |  |  |  |  |  |  |  |  |  | **X** |  |  |
| 5/31/15 | Turkey |  |  |  |  |  |  |  |  |  |  |  |  |  | **X** |  |  |  |  |  | **X** |  |
| 6/1/15 | Pullets |  |  |  |  | **X** |  |  |  |  |  |  |  |  | **X** |  |  |  |  |  |  |  |
| 6/1/15 | Turkey |  |  |  |  |  |  |  |  |  |  |  |  |  |  |  |  |  |  |  |  | **X** |
| 6/2/15 | Turkey |  |  |  |  |  |  |  |  |  |  |  |  |  |  |  |  |  |  |  |  |  |
| 6/2/15 | Turkey |  |  |  |  |  | **X** |  |  |  |  |  |  |  |  |  | **X** |  |  |  |  |  |
| 6/4/15 | Hatchery |  |  |  |  |  |  |  | **X** |  | **X** |  |  | **X** |  |  |  |  | **X** |  |  |  |
| 6/15/15 | Layer |  |  |  |  |  |  |  |  |  |  |  |  |  |  |  |  |  |  |  |  |  |

| **Outbreak**  **date** | **Type** | **Kansas (3/13/2015)** | | | | | | | | | | | | | | | | | | | | |
| --- | --- | --- | --- | --- | --- | --- | --- | --- | --- | --- | --- | --- | --- | --- | --- | --- | --- | --- | --- | --- | --- | --- |
|  |  | d-1 | d-2 | d-3 | d-4 | d-5 | d-6 | d-7 | d-8 | d-9 | d-10 | d-11 | d-12 | d-13 | d-14 | d-15 | d-16 | d-17 | d-18 | d-19 | d-20 | d-21 |
| 4/12/15 | Turkey |  |  |  |  |  | **X** |  |  |  |  |  |  |  |  |  |  |  |  |  |  |  |
| 4/16/15 | Turkey |  |  |  |  |  |  |  |  |  |  |  |  |  |  |  |  |  |  |  |  |  |
| 4/19/15 | Layer |  |  |  |  |  |  |  |  |  |  |  |  |  |  |  |  |  |  |  |  |  |
| 4/24/15 | Layer |  |  |  |  |  |  |  |  |  |  |  |  |  |  |  |  |  |  |  |  |  |
| 4/24/15 | Layer |  |  |  |  |  |  |  |  |  |  |  |  |  |  |  |  |  |  |  |  |  |
| 4/25/15 | Turkey |  |  |  |  |  |  |  |  |  |  |  |  |  |  |  |  |  |  |  |  |  |
| 4/26/15 | Pullets |  |  |  |  |  |  |  |  |  |  |  |  |  |  |  |  |  |  |  |  |  |
| 4/26/15 | Layer |  |  |  |  |  |  |  |  |  |  |  |  |  |  |  |  |  |  |  |  |  |
| 4/27/15 | Turkey |  |  |  |  |  |  |  |  |  |  |  |  |  |  |  |  |  |  |  |  |  |
| 4/28/15 | Layer |  |  |  |  |  |  |  |  |  |  |  |  |  |  |  |  |  |  |  |  |  |
| 4/28/15 | Turkey |  |  |  |  |  |  |  |  |  |  |  |  |  |  |  |  |  |  |  |  |  |
| 4/28/15 | Breeder |  |  |  |  |  |  |  |  |  |  |  |  |  |  |  |  |  |  |  |  |  |
| 4/29/15 | Layer |  |  |  |  |  |  |  |  |  |  |  |  |  |  |  |  |  |  |  |  |  |
| 4/29/15 | Turkey |  |  |  |  |  |  |  |  |  |  |  |  |  |  |  |  |  |  |  |  |  |
| 4/29/15 | Layer |  |  |  |  |  |  |  |  |  |  |  |  |  |  |  |  |  |  |  |  |  |
| 4/29/15 | Turkey |  |  |  |  |  |  |  |  |  |  |  |  |  |  |  |  |  |  |  |  |  |
| 4/29/15 | Layer |  |  |  |  |  |  |  |  |  |  |  |  |  |  |  |  |  |  |  |  |  |
| 4/30/15 | Turkey |  |  |  |  |  |  |  |  |  |  |  |  |  |  |  |  |  |  |  |  |  |
| 4/30/15 | Layer |  |  |  |  |  |  |  |  |  |  |  |  |  |  |  |  |  |  |  |  |  |
| 4/30/15 | Turkey |  |  |  |  |  |  |  |  |  |  |  |  |  |  |  |  |  |  |  |  |  |
| 5/1/15 | Turkey |  |  |  |  |  |  |  |  |  |  |  |  |  |  |  |  |  |  |  |  |  |
| 5/1/15 | Backyard |  |  |  |  |  |  |  |  |  |  |  |  |  |  |  |  |  |  |  |  |  |
| 5/2/15 | Turkey |  |  |  |  |  |  |  |  |  |  |  |  |  |  |  |  |  |  |  |  |  |
| 5/2/15 | Layer |  |  |  |  |  |  |  |  |  |  |  |  |  |  |  |  |  |  |  |  |  |
| 5/3/15 | Turkey |  |  |  |  |  |  |  |  |  |  |  |  |  |  |  |  |  |  |  |  |  |
| 5/3/15 | Layer |  |  |  |  |  |  |  |  |  |  |  |  |  |  |  |  |  |  |  |  |  |
| 5/3/15 | Layer |  |  |  |  |  |  |  |  |  |  |  |  |  |  |  |  |  |  |  |  |  |
| 5/4/15 | Pullets |  |  |  |  |  |  |  |  |  |  |  |  |  |  |  |  |  |  |  |  |  |
| 5/4/15 | Layer |  |  |  |  |  |  |  |  |  |  |  |  |  |  |  |  |  |  |  |  |  |
| 5/5/15 | Turkey |  |  |  |  |  |  |  |  |  |  |  |  |  |  |  |  |  |  |  |  |  |
| 5/5/15 | Turkey |  |  |  |  |  |  |  |  |  |  |  |  |  |  |  |  |  |  |  |  |  |
| 5/5/15 | Pullets |  |  |  |  |  |  |  |  |  |  |  |  |  |  |  |  |  |  |  |  |  |
| 5/5/15 | Pullets |  |  |  |  |  |  |  |  |  |  |  |  |  |  |  |  |  |  |  |  |  |
| 5/5/15 | Pullets |  |  |  |  |  |  |  |  |  |  |  |  |  |  |  |  |  |  |  |  |  |
| 5/5/15 | Layer |  |  |  |  |  |  |  |  |  |  |  |  |  |  |  |  |  |  |  |  |  |
| 5/6/15 | Turkey |  |  |  |  |  |  |  |  |  |  |  |  |  |  |  |  |  |  |  |  |  |
| 5/6/15 | Pullets |  |  |  |  |  |  |  |  |  |  |  |  |  |  |  |  |  |  |  |  |  |
| 5/6/15 | Turkey |  |  |  |  |  |  |  |  |  |  |  |  |  |  |  |  |  |  |  |  |  |
| 5/6/15 | Turkey |  |  |  |  |  |  |  |  |  |  |  |  |  |  |  |  |  |  |  |  |  |
| 5/7/15 | Turkey |  |  |  |  |  |  |  |  |  |  |  |  |  |  |  |  |  |  |  |  |  |
| 5/7/15 | Turkey |  |  |  |  |  |  |  |  |  |  |  |  |  |  |  |  |  |  |  |  |  |
| 5/7/15 | Backyard |  |  |  |  |  |  |  |  |  |  |  |  |  |  |  |  |  |  |  |  |  |
| 5/7/15 | Layer |  |  |  |  |  |  |  |  |  |  |  |  |  |  |  |  |  |  |  |  |  |
| 5/8/15 | Turkey |  |  |  |  |  |  |  |  |  |  |  |  |  |  |  |  |  |  |  |  |  |
| 5/9/15 | Pullets |  |  |  |  |  |  |  |  |  |  |  |  |  |  |  |  |  |  |  |  |  |
| 5/10/15 | Turkey |  |  |  |  |  |  |  |  |  |  |  |  |  |  |  |  |  |  |  |  |  |
| 5/10/15 | Turkey |  |  |  |  |  |  |  |  |  |  |  |  |  |  |  |  |  |  |  |  |  |
| 5/11/15 | Pullets |  |  |  |  |  |  |  |  |  |  |  |  |  |  |  |  |  |  |  |  |  |
| 5/11/15 | Layer |  |  |  |  |  |  |  |  |  |  |  |  |  |  |  |  |  |  |  |  |  |
| 5/12/15 | Pullets |  |  |  |  |  |  |  |  |  |  |  |  |  |  |  |  |  |  |  |  |  |
| 5/12/15 | Layer |  |  |  |  |  |  |  |  |  |  |  |  |  |  |  |  |  |  |  |  |  |
| 5/12/15 | Pullets |  |  |  |  |  |  |  |  |  |  |  |  |  |  |  |  |  |  |  |  |  |
| 5/13/15 | Backyard |  |  |  |  |  |  |  |  |  |  |  |  |  |  |  |  |  |  |  |  |  |
| 5/14/15 | Backyard |  |  |  |  |  |  |  |  |  |  |  |  |  |  |  |  |  |  |  |  |  |
| 5/14/15 | Backyard |  |  |  |  |  |  |  |  |  |  |  |  |  |  |  |  |  |  |  |  |  |
| 5/15/15 | Turkey |  |  |  |  |  |  |  |  |  |  |  |  |  |  |  |  |  |  |  |  |  |
| 5/17/15 | Turkey |  |  |  |  |  |  |  |  |  |  |  |  |  |  |  |  |  |  |  |  |  |
| 5/18/15 | Pullets |  |  |  |  |  |  |  |  |  |  |  |  |  |  |  |  |  |  |  |  |  |
| 5/18/15 | Layer |  |  |  |  |  |  |  |  |  |  |  |  |  |  |  |  |  |  |  |  |  |
| 5/19/15 | Turkey |  |  |  |  |  |  |  |  |  |  |  |  |  |  |  |  |  |  |  |  |  |
| 5/19/15 | Turkey |  |  |  |  |  |  |  |  |  |  |  |  |  |  |  |  |  |  |  |  |  |
| 5/19/15 | Turkey |  |  |  |  |  |  |  |  |  |  |  |  |  |  |  |  |  |  |  |  |  |
| 5/20/15 | Turkey |  |  |  |  |  |  |  |  |  |  |  |  |  |  |  |  |  |  |  |  |  |
| 5/24/15 | Layer |  |  |  |  |  |  |  |  |  |  |  |  |  |  |  |  |  |  |  |  |  |
| 5/25/15 | Layer |  |  |  |  |  |  |  |  |  |  |  |  |  |  |  |  |  |  |  |  |  |
| 5/25/15 | Layer |  |  |  |  |  |  |  |  |  |  |  |  |  |  |  |  |  |  |  |  |  |
| 5/27/15 | Turkey |  |  |  |  |  |  |  |  |  |  |  |  |  |  |  |  |  |  |  |  |  |
| 5/27/15 | Pullets |  |  |  |  |  |  |  |  |  |  |  |  |  |  |  |  |  |  |  |  |  |
| 5/28/15 | Turkey |  |  |  |  |  |  |  |  |  |  |  |  |  |  |  |  |  |  |  |  |  |
| 5/29/15 | Turkey |  |  |  |  |  |  |  |  |  |  |  |  |  |  |  |  |  |  |  |  |  |
| 5/31/15 | Turkey |  |  |  |  |  |  |  |  |  |  |  |  |  |  |  |  |  |  |  |  |  |
| 6/1/15 | Pullets |  |  |  |  |  |  |  |  |  |  |  |  |  |  |  |  |  |  |  |  |  |
| 6/1/15 | Turkey |  |  |  |  |  |  |  |  |  |  |  |  |  |  |  |  |  |  |  |  |  |
| 6/2/15 | Turkey |  |  |  |  |  |  |  |  |  |  |  |  |  |  |  |  |  |  |  |  |  |
| 6/2/15 | Turkey |  |  |  |  |  |  |  |  |  |  |  |  |  |  |  |  |  |  |  |  |  |
| 6/4/15 | Hatchery |  |  |  |  |  |  |  |  |  |  |  |  |  |  |  |  |  |  |  |  |  |
| 6/15/15 | Layer |  |  |  |  |  |  |  |  |  |  |  |  |  |  |  |  |  |  |  |  |  |

| **Outbreak**  **date** | **Type** | **North Dakota (4/10/2015-4/24/2015)** | | | | | | | | | | | | | | | | | | | | |
| --- | --- | --- | --- | --- | --- | --- | --- | --- | --- | --- | --- | --- | --- | --- | --- | --- | --- | --- | --- | --- | --- | --- |
|  |  | d-1 | d-2 | d-3 | d-4 | d-5 | d-6 | d-7 | d-8 | d-9 | d-10 | d-11 | d-12 | d-13 | d-14 | d-15 | d-16 | d-17 | d-18 | d-19 | d-20 | d-21 |
| 4/12/15 | Turkey |  |  |  |  |  |  |  |  |  |  |  |  |  |  |  |  |  |  |  |  |  |
| 4/16/15 | Turkey |  |  |  |  |  |  |  |  |  |  |  |  |  |  |  |  |  |  |  |  |  |
| 4/19/15 | Layer |  |  |  |  |  |  | **X** |  |  |  |  |  |  |  |  |  |  |  |  |  |  |
| 4/24/15 | Layer |  |  | **X** |  | **X** |  |  |  |  |  |  |  |  |  |  |  |  |  |  |  |  |
| 4/24/15 | Layer |  |  |  |  |  |  |  |  |  |  |  |  |  |  |  |  |  |  |  |  |  |
| 4/25/15 | Turkey |  |  |  |  |  |  |  |  |  |  |  |  |  |  |  |  |  |  |  |  |  |
| 4/26/15 | Pullets |  |  |  |  |  |  |  |  |  |  |  |  |  |  |  |  |  |  |  |  |  |
| 4/26/15 | Layer |  |  |  |  |  |  |  |  |  |  |  |  |  |  |  |  |  |  |  |  |  |
| 4/27/15 | Turkey |  |  |  |  |  |  |  |  |  |  |  |  |  |  |  |  |  |  |  |  |  |
| 4/28/15 | Layer |  |  |  |  |  |  |  |  |  |  |  |  |  |  |  |  |  |  |  |  |  |
| 4/28/15 | Turkey |  |  |  |  |  |  |  |  |  |  |  |  |  |  |  |  |  |  |  |  |  |
| 4/28/15 | Breeder |  |  |  |  |  |  |  |  |  |  |  |  |  |  |  |  |  |  |  |  |  |
| 4/29/15 | Layer |  |  |  |  |  |  |  |  |  |  |  |  |  |  |  |  |  |  |  |  |  |
| 4/29/15 | Turkey |  |  |  |  |  |  |  |  |  |  |  |  |  |  |  |  |  |  |  |  |  |
| 4/29/15 | Layer |  |  |  |  |  |  |  |  |  |  |  |  |  |  |  |  |  |  |  |  |  |
| 4/29/15 | Turkey |  |  |  |  |  |  |  |  |  |  |  |  |  |  |  |  |  |  |  |  |  |
| 4/29/15 | Layer |  |  |  |  |  |  |  |  |  |  |  |  |  |  |  |  |  |  |  |  |  |
| 4/30/15 | Turkey |  |  |  |  |  |  |  |  |  |  |  |  |  |  |  |  |  |  |  |  |  |
| 4/30/15 | Layer |  |  |  |  |  |  |  |  |  |  |  |  |  |  |  |  |  |  |  |  |  |
| 4/30/15 | Turkey |  |  |  |  |  |  |  |  |  |  |  |  |  |  |  |  |  |  |  |  |  |
| 5/1/15 | Turkey |  |  |  |  |  |  |  |  |  |  |  |  |  |  |  |  |  |  |  |  |  |
| 5/1/15 | Backyard |  |  |  |  |  |  |  |  |  |  |  |  |  |  |  |  |  |  |  |  |  |
| 5/2/15 | Turkey |  |  |  |  |  |  |  |  |  |  |  |  |  |  |  |  |  |  |  |  |  |
| 5/2/15 | Layer |  |  |  |  |  |  |  |  |  |  |  |  |  |  |  |  |  |  |  |  |  |
| 5/3/15 | Turkey |  |  |  |  |  |  |  |  |  |  |  |  |  |  |  |  |  |  |  |  |  |
| 5/3/15 | Layer |  |  |  |  |  |  |  |  |  |  |  |  |  |  |  |  |  |  |  |  |  |
| 5/3/15 | Layer |  |  |  |  |  |  |  |  |  |  |  |  |  |  |  |  |  |  |  |  |  |
| 5/4/15 | Pullets |  |  |  |  |  |  |  |  |  |  |  |  |  |  |  |  |  |  |  |  |  |
| 5/4/15 | Layer |  |  |  |  |  |  |  |  |  |  |  |  |  |  |  |  |  |  |  |  |  |
| 5/5/15 | Turkey |  |  |  |  |  |  |  |  |  |  |  |  |  |  |  |  |  |  |  |  |  |
| 5/5/15 | Turkey |  |  |  |  |  |  |  |  |  |  |  |  |  |  |  |  |  |  |  |  |  |
| 5/5/15 | Pullets |  |  |  |  |  |  |  |  |  |  |  |  |  |  |  |  |  |  |  |  |  |
| 5/5/15 | Pullets |  |  |  |  |  |  |  |  |  |  |  |  |  |  |  |  |  |  |  |  |  |
| 5/5/15 | Pullets |  |  |  |  |  |  |  |  |  |  |  |  |  |  |  |  |  |  |  |  |  |
| 5/5/15 | Layer |  |  |  |  |  |  |  |  |  |  |  |  |  |  |  |  |  |  |  |  |  |
| 5/6/15 | Turkey |  |  |  |  |  |  |  |  |  |  |  |  |  |  |  |  |  |  |  |  |  |
| 5/6/15 | Pullets |  |  |  |  |  |  |  |  |  |  |  |  |  |  |  |  |  |  |  |  |  |
| 5/6/15 | Turkey |  |  |  |  |  |  |  |  |  |  |  |  |  |  |  |  |  |  |  |  |  |
| 5/6/15 | Turkey |  |  |  |  |  |  |  |  |  |  |  |  |  |  |  |  |  |  |  |  |  |
| 5/7/15 | Turkey |  |  |  |  |  |  |  |  |  |  |  |  |  |  |  |  |  |  |  |  |  |
| 5/7/15 | Turkey |  |  |  |  |  |  |  |  |  |  |  |  |  |  |  |  |  |  |  |  |  |
| 5/7/15 | Backyard |  |  |  |  |  |  |  |  |  |  |  |  |  |  |  |  |  |  |  |  |  |
| 5/7/15 | Layer |  |  |  |  |  |  |  |  |  |  |  |  |  |  |  |  |  |  |  |  |  |
| 5/8/15 | Turkey |  |  |  |  |  |  |  |  |  |  |  |  |  |  |  |  |  |  |  |  |  |
| 5/9/15 | Pullets |  |  |  |  |  |  |  |  |  |  |  |  |  |  |  |  |  |  |  |  |  |
| 5/10/15 | Turkey |  |  |  |  |  |  |  |  |  |  |  |  |  |  |  |  |  |  |  |  |  |
| 5/10/15 | Turkey |  |  |  |  |  |  |  |  |  |  |  |  |  |  |  |  |  |  |  |  |  |
| 5/11/15 | Pullets |  |  |  |  |  |  |  |  |  |  |  |  |  |  |  |  |  |  |  |  |  |
| 5/11/15 | Layer |  |  |  |  |  |  |  |  |  |  |  |  |  |  |  |  |  |  |  |  |  |
| 5/12/15 | Pullets |  |  |  |  |  |  |  |  |  |  |  |  |  |  |  |  |  |  |  |  |  |
| 5/12/15 | Layer |  |  |  |  |  |  |  |  |  |  |  |  |  |  |  |  |  |  |  |  |  |
| 5/12/15 | Pullets |  |  |  |  |  |  |  |  |  |  |  |  |  |  |  |  |  |  |  |  |  |
| 5/13/15 | Backyard |  |  |  |  |  |  |  |  |  |  |  |  |  |  |  |  |  |  |  |  |  |
| 5/14/15 | Backyard |  |  |  |  |  |  |  |  |  |  |  |  |  |  |  |  |  |  |  |  |  |
| 5/14/15 | Backyard |  |  |  |  |  |  |  |  |  |  |  |  |  |  |  |  |  |  |  |  |  |
| 5/15/15 | Turkey |  |  |  |  |  |  |  |  |  |  |  |  |  |  |  |  |  |  |  |  |  |
| 5/17/15 | Turkey |  |  |  |  |  |  |  |  |  |  |  |  |  |  |  |  |  |  |  |  |  |
| 5/18/15 | Pullets |  |  |  |  |  |  |  |  |  |  |  |  |  |  |  |  |  |  |  |  |  |
| 5/18/15 | Layer |  |  |  |  |  |  |  |  |  |  |  |  |  |  |  |  |  |  |  |  |  |
| 5/19/15 | Turkey |  |  |  |  |  |  |  |  |  |  |  |  |  |  |  |  |  |  |  |  |  |
| 5/19/15 | Turkey |  |  |  |  |  |  |  |  |  |  |  |  |  |  |  |  |  |  |  |  |  |
| 5/19/15 | Turkey |  |  |  |  |  |  |  |  |  |  |  |  |  |  |  |  |  |  |  |  |  |
| 5/20/15 | Turkey |  |  |  |  |  |  |  |  |  |  |  |  |  |  |  |  |  |  |  |  |  |
| 5/24/15 | Layer |  |  |  |  |  |  |  |  |  |  |  |  |  |  |  |  |  |  |  |  |  |
| 5/25/15 | Layer |  |  |  |  |  |  |  |  |  |  |  |  |  |  |  |  |  |  |  |  |  |
| 5/25/15 | Layer |  |  |  |  |  |  |  |  |  |  |  |  |  |  |  |  |  |  |  |  |  |
| 5/27/15 | Turkey |  |  |  |  |  |  |  |  |  |  |  |  |  |  |  |  |  |  |  |  |  |
| 5/27/15 | Pullets |  |  |  |  |  |  |  |  |  |  |  |  |  |  |  |  |  |  |  |  |  |
| 5/28/15 | Turkey |  |  |  |  |  |  |  |  |  |  |  |  |  |  |  |  |  |  |  |  |  |
| 5/29/15 | Turkey |  |  |  |  |  |  |  |  |  |  |  |  |  |  |  |  |  |  |  |  |  |
| 5/31/15 | Turkey |  |  |  |  |  |  |  |  |  |  |  |  |  |  |  |  |  |  |  |  |  |
| 6/1/15 | Pullets |  |  |  |  |  |  |  |  |  |  |  |  |  |  |  |  |  |  |  |  |  |
| 6/1/15 | Turkey |  |  |  |  |  |  |  |  |  |  |  |  |  |  |  |  |  |  |  |  |  |
| 6/2/15 | Turkey |  |  |  |  |  |  |  |  |  |  |  |  |  |  |  |  |  |  |  |  |  |
| 6/2/15 | Turkey |  |  |  |  |  |  |  |  |  |  |  |  |  |  |  |  |  |  |  |  |  |
| 6/4/15 | Hatchery |  |  |  |  |  |  |  |  |  |  |  |  |  |  |  |  |  |  |  |  |  |
| 6/15/15 | Layer |  |  |  |  |  |  |  |  |  |  |  |  |  |  |  |  |  |  |  |  |  |

| **Outbreak**  **date** | **Type** | **Iowa (4/12/2015-6/15/2015)** | | | | | | | | | | | | | | | | | | | | |
| --- | --- | --- | --- | --- | --- | --- | --- | --- | --- | --- | --- | --- | --- | --- | --- | --- | --- | --- | --- | --- | --- | --- |
|  |  | d-1 | d-2 | d-3 | d-4 | d-5 | d-6 | d-7 | d-8 | d-9 | d-10 | d-11 | d-12 | d-13 | d-14 | d-15 | d-16 | d-17 | d-18 | d-19 | d-20 | d-21 |
| 4/12/15 | Turkey |  |  |  |  |  |  |  |  |  |  |  |  |  |  |  |  |  |  |  |  |  |
| 4/16/15 | Turkey |  |  |  |  |  |  |  |  |  |  |  |  |  |  |  |  |  |  |  |  |  |
| 4/19/15 | Layer |  |  |  |  | **X** |  |  |  |  |  |  |  |  |  |  |  |  |  |  |  |  |
| 4/24/15 | Layer |  |  |  |  |  |  | **X** |  | **X** |  |  |  |  |  |  |  |  |  |  |  |  |
| 4/24/15 | Layer | **X** |  |  |  |  | **X** | **X** |  | **X** |  |  |  |  |  |  |  |  |  |  |  |  |
| 4/25/15 | Turkey |  |  |  | **X** |  |  |  |  | **X** |  |  |  | **X** | **X** |  |  |  |  |  |  |  |
| 4/26/15 | Pullets |  | **X** | **X** |  |  |  |  | **X** |  |  |  |  |  |  |  |  |  |  |  |  |  |
| 4/26/15 | Layer | **X** |  | **X** |  |  |  |  | **X** |  |  |  |  |  |  |  |  |  |  |  |  |  |
| 4/27/15 | Turkey |  |  | **X** |  |  |  |  |  |  |  |  |  |  |  | **X** | **X** |  |  |  |  |  |
| 4/28/15 | Layer |  |  |  | **X** |  |  |  |  |  |  |  |  |  |  |  |  | **X** |  |  |  |  |
| 4/28/15 | Turkey | **X** | **X** |  |  |  |  |  |  |  |  |  | **X** |  |  |  |  |  |  |  |  |  |
| 4/28/15 | Breeder |  |  |  |  |  |  | **X** |  |  |  |  |  |  |  |  | **X** |  |  |  |  |  |
| 4/29/15 | Layer | **X** |  |  |  |  |  |  |  |  |  |  |  |  |  |  |  |  |  |  |  |  |
| 4/29/15 | Turkey | **X** |  |  |  |  |  | **X** |  |  |  |  |  | **X** |  |  |  |  |  |  |  |  |
| 4/29/15 | Layer | **X** |  |  |  | **X** |  |  |  |  |  |  |  |  |  | **X** |  |  |  |  |  |  |
| 4/29/15 | Turkey | **X** | **X** | **X** | **X** |  |  |  | **X** |  | **X** |  |  | **X** |  | **X** |  | **X** |  | **X** | **X** |  |
| 4/29/15 | Layer |  | **X** | **X** | **X** | **X** | **X** |  |  |  |  | **X** |  |  |  |  |  |  |  |  |  |  |
| 4/30/15 | Turkey | **X** | **X** | **X** |  |  |  |  |  | **X** |  | **X** | **X** |  |  |  | **X** |  |  | **X** | **X** | **X** |
| 4/30/15 | Layer | **X** | **X** |  |  |  |  |  |  |  |  |  |  |  |  |  |  |  |  |  |  |  |
| 4/30/15 | Turkey |  | **X** |  |  |  |  |  |  |  |  |  |  | **X** |  |  |  |  |  |  |  |  |
| 5/1/15 | Turkey | **X** | **X** | **X** | **X** |  |  | **X** |  |  |  |  |  | **X** |  |  | **X** |  |  |  |  |  |
| 5/1/15 | Backyard | **X** | **X** |  | **X** | **X** | **X** | **X** | **X** |  |  |  |  | **X** |  |  | **X** |  |  |  |  |  |
| 5/2/15 | Turkey | **X** | **X** | **X** | **X** | **X** |  |  | **X** |  |  | **X** |  | **X** | **X** |  | **X** |  |  | **X** | **X** | **X** |
| 5/2/15 | Layer | **X** | **X** | **X** |  |  | **X** | **X** | **X** | **X** |  |  |  |  | **X** |  |  | **X** |  |  |  |  |
| 5/3/15 | Turkey |  |  | **X** | **X** | **X** | **X** | **X** | **X** |  |  |  |  |  |  | **X** | **X** | **X** |  |  |  |  |
| 5/3/15 | Layer |  |  | **X** | **X** | **X** | **X** | **X** | **X** |  |  | **X** |  |  |  | **X** |  |  |  |  |  | **X** |
| 5/3/15 | Layer |  |  | **X** | **X** |  | **X** | **X** |  | **X** | **X** |  |  |  |  | **X** |  |  |  |  |  |  |
| 5/4/15 | Pullets | **X** | **X** |  | **X** | **X** |  |  | **X** | **X** | **X** | **X** |  |  |  |  | **X** |  |  |  | **X** |  |
| 5/4/15 | Layer | **X** |  |  |  | **X** | **X** |  |  |  |  |  | **X** | **X** | **X** | **X** |  |  |  |  |  |  |
| 5/5/15 | Turkey | **X** |  |  |  | **X** | **X** | **X** | **X** | **X** | **X** |  |  |  |  |  |  | **X** | **X** |  | **X** |  |
| 5/5/15 | Turkey | **X** | **X** |  |  | **X** | **X** | **X** |  |  |  |  |  | **X** | **X** |  | **X** |  |  |  | **X** |  |
| 5/5/15 | Pullets | **X** | **X** |  |  | **X** | **X** | **X** | **X** | **X** | **X** | **X** | **X** |  | **X** |  |  | **X** |  |  |  |  |
| 5/5/15 | Pullets | **X** | **X** |  |  | **X** | **X** |  | **X** | **X** | **X** |  | **X** |  |  |  |  | **X** |  |  |  |  |
| 5/5/15 | Pullets | **X** | **X** |  |  |  | **X** |  | **X** | **X** | **X** | **X** |  |  |  |  |  | **X** |  |  |  |  |
| 5/5/15 | Layer | **X** | **X** |  |  | **X** | **X** |  | **X** | **X** | **X** | **X** |  | **X** |  |  |  | **X** |  |  |  | **X** |
| 5/6/15 | Turkey | **X** | **X** | **X** | **X** | **X** | **X** |  | **X** | **X** | **X** | **X** |  |  |  |  |  |  |  | **X** |  | **X** |
| 5/6/15 | Pullets | **X** |  | **X** | **X** | **X** | **X** |  |  |  |  |  | **X** | **X** |  |  |  |  |  | **X** |  |  |
| 5/6/15 | Turkey | **X** | **X** | **X** |  |  | **X** | **X** | **X** |  |  |  |  |  | **X** | **X** | **X** |  | **X** |  |  |  |
| 5/6/15 | Turkey | **X** | **X** | **X** |  |  | **X** | **X** | **X** | **X** |  |  |  |  |  |  |  |  |  | **X** | **X** |  |
| 5/7/15 | Turkey | **X** | **X** | **X** | **X** |  |  | **X** | **X** | **X** | **X** | **X** | **X** |  |  |  |  |  |  |  |  | **X** |
| 5/7/15 | Turkey | **X** | **X** | **X** | **X** |  |  | **X** | **X** | **X** | **X** | **X** | **X** |  |  |  |  |  |  |  | **X** |  |
| 5/7/15 | Backyard | **X** | **X** |  | **X** | **X** | **X** | **X** | **X** | **X** | **X** | **X** | **X** | **X** | **X** | **X** | **X** | **X** | **X** |  | **X** |  |
| 5/7/15 | Layer |  |  |  | **X** |  |  |  | **X** | **X** |  |  |  |  |  |  | **X** | **X** | **X** |  |  |  |
| 5/8/15 | Turkey | **X** | **X** | **X** | **X** | **X** | **X** | **X** |  | **X** | **X** | **X** | **X** | **X** | **X** | **X** |  |  |  |  |  |  |
| 5/9/15 | Pullets | **X** | **X** |  |  |  | **X** |  |  | **X** | **X** |  |  |  |  |  | **X** | **X** | **X** |  | **X** |  |
| 5/10/15 | Turkey | **X** | **X** | **X** | **X** | **X** | **X** | **X** |  | **X** | **X** | **X** |  |  | **X** |  | **X** |  |  |  | **X** |  |
| 5/10/15 | Turkey | **X** | **X** | **X** | **X** | **X** | **X** | **X** | **X** | **X** | **X** |  |  |  | **X** | **X** |  | **X** |  |  |  | **X** |
| 5/11/15 | Pullets | **X** |  | **X** | **X** | **X** | **X** | **X** | **X** |  |  | **X** | **X** |  |  |  |  | **X** |  |  |  | **X** |
| 5/11/15 | Layer | **X** | **X** |  | **X** | **X** | **X** | **X** | **X** | **X** | **X** | **X** | **X** |  |  |  | **X** | **X** | **X** | **X** |  |  |
| 5/12/15 | Pullets | **X** | **X** | **X** | **X** | **X** | **X** | **X** |  | **X** | **X** |  |  | **X** |  | **X** | **X** | **X** |  |  |  |  |
| 5/12/15 | Layer | **X** | **X** | **X** |  | **X** | **X** | **X** | **X** | **X** | **X** |  | **X** | **X** | **X** | **X** | **X** | **X** |  |  | **X** |  |
| 5/12/15 | Pullets |  | **X** | **X** | **X** |  |  | **X** | **X** | **X** | **X** |  | **X** | **X** | **X** | **X** | **X** | **X** |  |  | **X** |  |
| 5/13/15 | Backyard | **X** |  | **X** | **X** | **X** | **X** | **X** | **X** | **X** | **X** | **X** | **X** | **X** | **X** | **X** | **X** | **X** | **X** | **X** | **X** | **X** |
| 5/14/15 | Backyard | **X** | **X** | **X** | **X** | **X** | **X** | **X** | **X** | **X** | **X** | **X** | **X** | **X** | **X** | **X** | **X** | **X** | **X** | **X** | **X** | **X** |
| 5/14/15 | Backyard | **X** | **X** | **X** | **X** | **X** | **X** | **X** | **X** | **X** | **X** | **X** | **X** | **X** | **X** | **X** | **X** | **X** | **X** | **X** | **X** | **X** |
| 5/15/15 | Turkey | **X** | **X** | **X** | **X** | **X** | **X** | **X** |  | **X** | **X** | **X** | **X** |  | **X** |  | **X** | **X** | **X** | **X** |  |  |
| 5/17/15 | Turkey |  | **X** | **X** | **X** | **X** | **X** | **X** | **X** | **X** | **X** | **X** | **X** | **X** | **X** |  |  | **X** | **X** | **X** | **X** | **X** |
| 5/18/15 | Pullets | **X** | **X** | **X** | **X** | **X** |  | **X** | **X** | **X** | **X** | **X** | **X** | **X** | **X** | **X** | **X** |  | **X** | **X** | **X** | **X** |
| 5/18/15 | Layer | **X** | **X** | **X** | **X** | **X** | **X** | **X** | **X** | **X** | **X** | **X** | **X** | **X** | **X** | **X** |  |  | **X** | **X** |  |  |
| 5/19/15 | Turkey | **X** | **X** |  |  |  |  | **X** | **X** | **X** | **X** |  | **X** | **X** | **X** |  |  |  |  | **X** | **X** | **X** |
| 5/19/15 | Turkey | **X** | **X** |  |  |  | **X** | **X** | **X** | **X** | **X** | **X** |  | **X** | **X** | **X** | **X** |  |  | **X** | **X** |  |
| 5/19/15 | Turkey | **X** | **X** | **X** | **X** | **X** | **X** | **X** | **X** | **X** | **X** | **X** | **X** | **X** | **X** | **X** | **X** |  |  | **X** | **X** | **X** |
| 5/20/15 | Turkey | **X** | **X** | **X** |  |  | **X** |  | **X** | **X** | **X** | **X** | **X** | **X** | **X** | **X** |  |  |  |  | **X** | **X** |
| 5/24/15 | Layer |  |  |  | **X** | **X** | **X** |  |  |  | **X** |  |  |  |  | **X** |  | **X** |  |  | **X** |  |
| 5/25/15 | Layer |  |  | **X** | **X** | **X** |  | **X** | **X** |  |  | **X** |  |  | **X** | **X** |  |  | **X** | **X** | **X** |  |
| 5/25/15 | Layer | **X** |  | **X** | **X** |  |  | **X** | **X** |  |  |  | **X** |  |  |  |  |  |  | **X** |  |  |
| 5/27/15 | Turkey | **X** |  |  |  |  | **X** |  | **X** | **X** |  |  |  |  |  | **X** | **X** |  |  | **X** | **X** |  |
| 5/27/15 | Pullets | **X** |  |  |  | **X** | **X** | **X** |  | **X** | **X** |  | **X** |  |  |  | **X** | **X** |  |  | **X** |  |
| 5/28/15 | Turkey |  | **X** |  |  |  |  | **X** | **X** | **X** | **X** | **X** |  | **X** | **X** | **X** | **X** | **X** | **X** | **X** | **X** |  |
| 5/29/15 | Turkey |  | **X** | **X** |  |  |  | **X** | **X** |  |  | **X** |  |  |  |  |  | **X** | **X** |  |  | **X** |
| 5/31/15 | Turkey |  | **X** | **X** | **X** | **X** |  |  |  |  | **X** | **X** | **X** | **X** | **X** | **X** | **X** | **X** |  | **X** | **X** | **X** |
| 6/1/15 | Pullets |  |  | **X** | **X** | **X** |  | **X** | **X** |  |  | **X** | **X** | **X** | **X** | **X** |  |  | **X** | **X** | **X** | **X** |
| 6/1/15 | Turkey | **X** |  | **X** |  | **X** | **X** |  |  |  | **X** | **X** |  |  | **X** |  |  |  |  |  | **X** | **X** |
| 6/2/15 | Turkey |  |  | **X** | **X** |  | **X** | **X** |  |  |  |  | **X** |  |  | **X** |  |  |  |  |  | **X** |
| 6/2/15 | Turkey |  | **X** | **X** | **X** | **X** |  |  |  |  |  | **X** | **X** |  |  | **X** | **X** |  |  |  | **X** | **X** |
| 6/4/15 | Hatchery | **X** |  |  | **X** |  | **X** |  | **X** |  |  | **X** | **X** | **X** |  |  |  |  |  | **X** | **X** | **X** |
| 6/15/15 | Layer |  |  |  |  |  | **X** |  |  |  |  |  |  |  |  |  |  | **X** | **X** |  | **X** |  |

**Table S3. Summary of 77 Iowa cases during 2015 HPAI outbreak.**

| Parameter | Value |
| --- | --- |
| **Total cases/farms** | 77 |
| **Type** | Turkey – 35 cases  Layer – 22 cases  Layer pullet – 13 cases  Backyard – 5 cases  Breeder – 1 case  Hatchery – 1 case |
| **First outbreak date** | 4/12/2015 (Turkey) |
| **Last outbreak date** | 6/15/2015 (Layer) |
| **Latitude (Mean±SD)** | 41.332 ± 0.363 |
| **Longitude (Mean±SD)** | -96.424 ± 0.810 |

**Movie S1. Concentration contour map for airborne virus carried by PM_10_ during 2015 HPAI outbreak. Default input values are used for concentration modeling. The blue area shows the virus concentration in a range of 10^-7^-10^-4^ 50% egg infective dose per cubic meter (EID_50_/m^3^). ‘T’ (green) represents turkey farm, ‘L’ (red) represents laying-hen farm, ‘B’ (white) represents breeder farm, ‘P’ (orange) represent pullet farm, ‘Y’ (purple) represent backyard farm, ‘H’ (yellow) represents hatchery farm.**

**Movie S2. Concentration contour map for airborne virus carried by PM_2.5_ during 2015 HPAI outbreak. Default input values are used for concentration modeling. The blue area shows the virus concentration in a range of 10^-7^-10^-4^ 50% egg infective dose per cubic meter (EID_50_/m^3^).** **‘T’ (green) represents turkey farm, ‘L’ (red) represents laying-hen farm, ‘B’ (white) represents breeder farm, ‘P’ (orange) represent pullet farm, ‘Y’ (purple) represent backyard farm, ‘H’ (yellow) represents hatchery farm.**

**References**

1. Shepherd TA*, et al.* (2015) Environmental assessment of three egg production systems—Part II. Ammonia, greenhouse gas, and particulate matter emissions. *Poult. Sci.* 94(3):534-543.

2. Li H*, et al.* (2011) Air emissions from tom and hen turkey houses in the US Midwest. *Trans. ASABE* 54(1):305-314.

3. Cambra-López M, Aarnink AJA, Zhao Y, Calvet S, & Torres AG (2010) Airborne particulate matter from livestock production systems: A review of an air pollution problem. *Environ. Pollut.* 158(1):1-17.

4. Rosenthal E, Schneider T, Büscher W, & Diekmann B (2007) Sedimentation of animal-specific dust particles in livestock houses. *Landtechnik* 62(2):102-103.

5. Lin JJ, Noll KE, & Holsen TM (1994) Dry deposition velocities as a function of particle size in the ambient atmosphere. *Aerosol Sci. Tech.* 20(3):239-252.

6. Shaman J & Kohn M (2009) Absolute humidity modulates influenza survival, transmission, and seasonality. *PNAS* 106(9):3243-3248.

7. Zhao Y, Aarnink AJA, de Jong MCM, & Groot Koerkamp PWG (2014) Airborne microorganisms from livestock production systems and their relation to dust. *Crit. Rev. Env. Sci. Tec.* 44(10):1071-1128.

8. Spackman E, Pantin-Jackwood MJ, Kapczynski DR, Swayne DE, & Suarez DL (2016) H5N2 Highly Pathogenic Avian Influenza Viruses from the US 2014-2015 outbreak have an unusually long pre-clinical period in turkeys. *BMC Vet. Res.* 12(1):260.

9. DeJesus E*, et al.* (2016) Changes in adaptation of H5N2 highly pathogenic avian influenza H5 clade 2.3.4.4 viruses in chickens and mallards. *Virology* 499:52-64.

10. Forrest HL, Kim J, & Webster RG (2010) Virus shedding and potential for interspecies waterborne transmission of highly pathogenic H5N1 influenza virus in sparrows and chickens. *J. Virol.* 84(7):3718-3720.

11. VanDalen KK, Franklin AB, Mooers NL, Sullivan HJ, & Shriner SA (2010) Shedding light on avian influenza H4N6 infection in mallards: modes of transmission and implications for surveillance. *PLoS One* 5(9):e12851.

12. Kurmi B*, et al.* (2013) Survivability of highly pathogenic avian influenza H5N1 virus in poultry faeces at different temperatures. *Indian J. Virol.* 24(2):272-277.

13. Julian RJ (1989) Lung volume of meat-type chickens. *Avian Dis.* 33(1):174-176.

14. Pampori ZA & Iqbal S (2007) Haematology, serum chemistry and electrocardiographic evaluation in native chicken of Kashmir. *Int. J. Poul. Sci.* 6(8):578-582.

15. Dickson JG (1992) *The wild turkey: biology and management* (Stackpole Books).

16. Zhao Y, Shepherd TA, Li H, & Xin H (2015) Environmental assessment of three egg production systems–Part I: Monitoring system and indoor air quality. *Poult. Sci.* 94(3):518-533.
